# Aided and Unaided Speech Perception by Older Hearing Impaired Listeners

**DOI:** 10.1371/journal.pone.0114922

**Published:** 2015-03-02

**Authors:** David L. Woods, Tanya Arbogast, Zoe Doss, Masood Younus, Timothy J. Herron, E. William Yund

**Affiliations:** 1 Human Cognitive Neurophysiology Laboratory, VANCHCS, 150 Muir Rd., Martinez, California, 95553, United States of America; 2 UC Davis Department of Neurology, 4860 Y St., Suite 3700, Sacramento, California, 95817, United States of America; 3 Center for Neurosciences, UC Davis, 1544 Newton Ct., Davis, California, 95616, United States of America; 4 UC Davis Center for Mind and Brain, 202 Cousteau Place, Suite 201, Davis, California, 95616, United States of America; University Of Cambridge, UNITED KINGDOM

## Abstract

The most common complaint of older hearing impaired (OHI) listeners is difficulty understanding speech in the presence of noise. However, tests of consonant-identification and sentence reception threshold (SeRT) provide different perspectives on the magnitude of impairment. Here we quantified speech perception difficulties in 24 OHI listeners in unaided and aided conditions by analyzing (1) consonant-identification thresholds and consonant confusions for 20 onset and 20 coda consonants in consonant-vowel-consonant (CVC) syllables presented at consonant-specific signal-to-noise (SNR) levels, and (2) SeRTs obtained with the Quick Speech in Noise Test (QSIN) and the Hearing in Noise Test (HINT). Compared to older normal hearing (ONH) listeners, nearly all unaided OHI listeners showed abnormal consonant-identification thresholds, abnormal consonant confusions, and reduced psychometric function slopes. Average elevations in consonant-identification thresholds exceeded 35 dB, correlated strongly with impairments in mid-frequency hearing, and were greater for hard-to-identify consonants. Advanced digital hearing aids (HAs) improved average consonant-identification thresholds by more than 17 dB, with significant HA benefit seen in 83% of OHI listeners. HAs partially normalized consonant-identification thresholds, reduced abnormal consonant confusions, and increased the slope of psychometric functions. Unaided OHI listeners showed much smaller elevations in SeRTs (mean 6.9 dB) than in consonant-identification thresholds and SeRTs in unaided listening conditions correlated strongly (r = 0.91) with identification thresholds of easily identified consonants. HAs produced minimal SeRT benefit (2.0 dB), with only 38% of OHI listeners showing significant improvement. HA benefit on SeRTs was accurately predicted (r = 0.86) by HA benefit on easily identified consonants. Consonant-identification tests can accurately predict sentence processing deficits and HA benefit in OHI listeners.

## Introduction

The primary complaint of patients with sensorineural hearing loss (SNHL) is difficulty in understanding speech in noise. Indeed, 91% of older hearing impaired (OHI) listeners report difficulty understanding speech in noise, even when wearing hearing aids (HAs) [1]. However, different speech-perception tests provide different perspectives on the magnitude of impairment: OHI listeners generally show larger threshold elevations in consonant-identification tests [2–5] than in sentence tests [6–9]. For example, Phatak et al. [10] found that the signal-to-noise ratio (SNR) needed by OHI listeners to identify consonants in noise was increased by more than 20 dB compared to the thresholds of young normal-hearing (YNH) listeners. In contrast, they found that sentence reception thresholds (SeRTs), measured with the Quick Speech in Noise (QSIN) test [11], were elevated by only about 4.5 dB. Phatak et al [10] is the only study to directly compare consonant-identification and SeRT tests in unaided OHI listeners. Similarly, only one study has compared HA benefit on consonant-identification and SeRTs in the same listeners: Hopkins et al [12] found greater HA benefit on consonant identification than SeRTs.

Although many previous studies have shown that consonant-identification thresholds are significantly elevated in unaided OHI listeners [4,5,10,13–19], the relationship between audiometric loss at particular frequencies and consonant-identification deficits remains incompletely understood. For example, Yoon et al. [20] analyzed the results from Phatak et al. [10] and found that the confusions of OHI listeners were not correlated with their audiometric loss at any frequency. In contrast, Bosman and Smoorenburg [21] and van Rooij and Plomp [22] found high correlations between consonant-identification deficits and the average pure tone audiogram (PTA) from 0.5–2.0 kHz.

In addition to audiometric loss, several other factors influence the magnitude of consonant-identification deficits in OHI listeners. First, consonant-identification thresholds show small elevations in older normal-hearing listeners (ONH) [23], in part because of deficits in temporal processing [24]. Thus, to analyze the contribution of audiometric loss in older listeners, it is necessary to compare speech processing in OHI and ONH listener groups. Second, previous studies of consonant-identification performance have generally used percent correct or rau measures. While such measures are adequate for evaluating overall performance, hit rates for individual consonants are influenced by response bias, so signal-detection metrics are to be preferred [25]. Third, the accuracy of consonant identification in OHI listeners is influenced by vowel nuclei in consonant-vowel (CV) and vowel-consonant (VC) syllables [16]. Thus, to fully characterize the effects of hearing loss on consonant-identification thresholds vowel influences must be taken into consideration.

Although most consonants in natural speech occur in multi-consonant syllables, previous studies of consonant confusions in OHI listeners have largely relied on CV syllables [10] or separate sets of CVs and VCs [16,26]. In the current study, we used the California Syllable Test (CaST) [25] which uses consonant-vowel-consonant (CVC) syllables. We anticipated that consonant-identification thresholds would be significantly elevated in OHI listeners relative to previously collected data from ONH listeners [23], and that the magnitude of threshold elevation would vary substantially for different consonants [10]. We also tested the hypotheses that consonant threshold elevations in OHI listeners might vary for onset and coda consonants [4], and for consonants presented in syllables containing different vowels [16].

### Sentence and consonant thresholds

SeRTs measure the signal-to-noise ratio (SNR) needed to accurately repeat sentence lists when mixed with concurrent speech-spectrum noise, as in the Hearing in Noise Test (HINT) [27], or when mixed with multi-talker babble, as is the case with the Quick Speech in Noise test (QSIN) [11]. SeRTs are typically elevated in OHI listeners with sloping high-frequency hearing losses by 2–10 dB on different tests. For example, Wilson et al. [28] found that unaided OHI listeners showed threshold elevations ranging from 5.6 dB on the HINT to 7.9 dB on the QSIN. However, some OHI listeners with significantly elevated audiometric thresholds had SeRTs within the normal range [6,28].

SeRT elevations are generally smaller and less reliably observed among OHI listeners than elevations in consonant-identification thresholds [10,18,29]. Sentence processing also depends on cognitive and semantic processing [30]. For example, Benichov et al. [31] used identical sentence-ending words and found that hearing loss had a large effect on word recognition when words were presented in neutral carrier phrases, but had little influence on word recognition when words were presented in high-context sentences. Other studies have also demonstrated that SeRT elevations in hearing-impaired listeners are larger for low- than high-context sentences [32], as, for example, in the Speech In Noise Test [33]. Moreover, sentence comprehension is also influenced by cognitive abilities including attention, working memory, and processing speed [34,35]. For example, van Rooij and Plomp [22] and Lunner [36] found that cognitive factors explained 30–40% of the variance in speech recognition performance in unaided OHI listeners.

The identification of consonants depends on the audibility of mid- and high-frequency acoustic cues that are directly related to the listener’s corresponding audiometric thresholds. In contrast, sentence comprehension depends on a broader range of cues, including low-frequency vowel [37] and intonation cues which are accurately processed by OHI listeners [15,38]. OHI listeners can also perceive supra-segmental stress and prosody cues [39] which convey information about grammar [40].

Average consonant-identification thresholds, particularly for more difficult consonants, are significantly higher than HINT and QSIN SeRTs, even in NH listeners. For example, in NH listeners, more than 50% of consonants cannot be identified at the SNRs used in SeRT testing [23,41]. Thus, while aging significantly increases consonant-identification thresholds in ONH listeners, these elevations are not accompanied by increases in SeRTs [23], suggesting that older listeners compensate for impaired consonant identification by an increased reliance on top-down semantic processing.

Although top-down semantic processing can compensate for impaired consonant identification in many circumstances, in noisy listening conditions some consonants must be identified in order understand words that permit semantic context to be established. Therefore, we expected significant correlations between the thresholds for the most easily-identified consonants and SeRTs in OHI listeners. We also hypothesized that correlations between audiometric thresholds and SeRTs would be primarily mediated through consonant-identification thresholds; i.e., the independent influence of audiometric thresholds would largely vanish in multiple regression analyses of SeRTs that included both audiometric thresholds and consonant-identification thresholds.

### Hearing-aid benefit on speech comprehension

Previous studies have compared HA benefit on consonant identification for HAs with different types of amplification and generally found significant variations in benefit [2,3,12,42–45]. In addition, previous studies comparing performance in unaided and aided listening conditions have generally reported significant HA benefit on consonant identification [46–49], with two recent exceptions [26,50]. Because of the less direct relationship between SeRTs and hearing function, we anticipated smaller and more variable HA benefit on SeRTs than on consonant-identification thresholds [6,51,52].

## Methods

### Ethics statement

All listeners gave informed written consent following procedures approved by the Institutional Review Board of the VA Northern California Health Care System (VANCHCS) and received financial compensation for their participation.

### Listeners

The OHI group was comprised of 24 male Veteran patients with mild-to-moderately-severe SNHL (age range 61–81 yrs, mean 70 yrs) who had been fitted by the VA Audiology Service with bilateral digital hearing aids. All OHI listeners had sloping audiometric losses, symmetrical within 15 dB at all frequencies in the two ears. The ONH group was comprised of 16 listeners (14 female, age range 60–79 yrs, mean 67 yrs) with normal hearing (thresholds ≤ 25 dB HL at 0.25–4 kHz), whose data have been reported in detail elsewhere [23]. All listeners were in good mental and physical health, had normal daily functioning, and spoke English as their native language. Listeners were excluded if they had a history of dementia, mild cognitive impairment, chronic alcoholism, drug abuse, neurological disorders, on-going treatment with ototoxic drugs or psychopharmaceutical agents, severe head trauma, or chronic recurrent disease. S1 Table provides additional information about listener age, HA models, and HA fitting characteristics. All of the HAs were advanced digital models with 17–20 channels that were fitted to manufacturer-specified settings. Of the 24 listeners, 22 used Phonak HAs (Exelia, Ambra, and Audeo) and two used HAs from GN Resound. All listeners had been using their HAs for at least six months prior to testing. Attack/release times (ms) for the HAs were short: 1/10 (n = 2), 1/50 (n = 15), 10/50 (n = 5) and 12/70 (n = 2). In addition, half of the HAs included non-linear frequency compression.

### Audiometric testing

**Fig 1:**
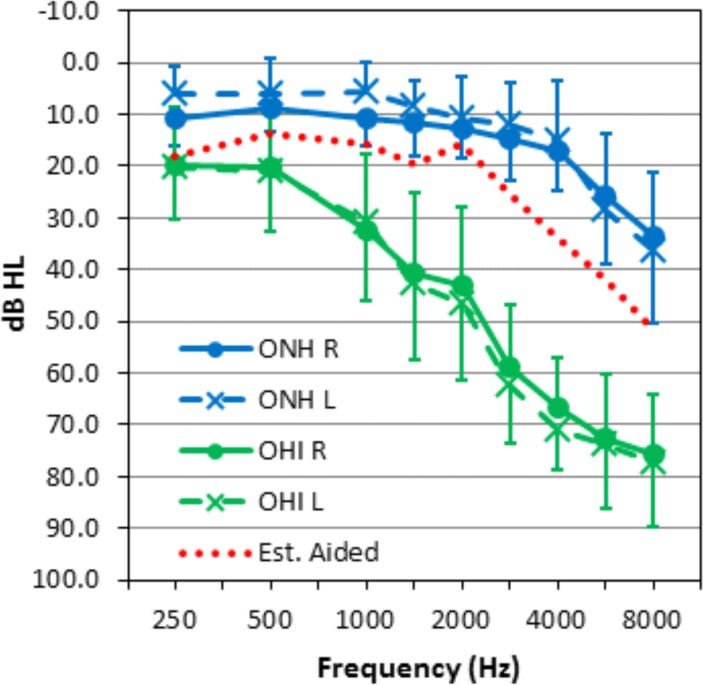
Average audiograms for 24 OHI listeners (green) and 16 ONH listeners (blue). Error bars show standard deviation. The dotted red line shows the estimated average aided audiogram based on prescriptive target for the average OHI audiogram.

Fig. 1 shows the average audiograms from the ONH and OHI groups: OHI listeners had normal hearing to mild losses at 0.5 kHz (maximum 40 dB), with losses increasing at higher frequencies to reach 70–80 dB at 8 kHz. The green line in Fig. 1 shows the estimated average audiometric thresholds in aided conditions, based on Phonak target settings for the average OHI audiometric thresholds.

### The California Syllable Test (CaST): stimuli

The CaST [25,41] includes 1,200 CVC syllables (9,600 tokens) constructed from the exhaustive combination of 20 onset consonants, 20 coda consonants, and 3 vowels (/ɑ/, /i/, /u/). Test materials and CaST software can be obtained at http://www.ebire.org/hcnlab/tools/hearing/CaST. Nineteen consonants (/p/, /t/, /k/, /f/, /ɵ/, /s/, /ʃ/, /ʧ/, /b/, /d/, /g/, /v/, /ð/, /z/, /ʤ/, /m/, /n/, /l/, and /r/) occur at onset and coda consonant positions, while /h/ occurs only in onset position and /ŋ/ only in coda. Based on previous studies of normal hearing (NH) listeners [23,25,41], these 21 consonants were divided into three groups based on the SNRs required for their identification. Group A consonants (/t/, /s/, /ʃ/, /ʧ/, /z/, /ʤ/, and /r/) are identified by NH listeners at the lowest SNRs, similar to the SNRs used in SeRT testing. Group B consonants (/k/, /f/, /d/, /g/, /m/, /n/, and /l/) are of intermediate difficulty, with most identified at SNRs 10–12 dB above SeRT SNRs. Group C consonants (/p/, /ɵ/, /b/, /v/, /h/, /ð/, and /ŋ/) are the most difficult to identify and have thresholds that are more than 20 dB above SeRT SNRs. Group A and B consonants are somewhat more common in conversational speech than Group C consonants. We quantified the incidence of most common American English consonants in film dialogs in the SUBTLEX data base and found that Group A consonants had an incidence of 39.85% (/t/ 11.73%, /s/ 8.06%, /ʃ/ 1.65%, /ʧ/ 0.95%, /z/ 4.68%, /ʤ/1.00%, and /r/ 11.78%), Group B consonants had an incidence of 40.63% (/k/ 5.40%, /f/ 2.90%, /d/ 7.15%, /g/ 1.36%, /m/ 4.68%, /n/ 12.07%, and /l/, 6.72%), and Group C consonants had an incidence of 19.88% (/p/ 3.65%, /ɵ/ 0.70%, /b/ 3.05%, /v/ 3.41%, /h/ 2.38%, /ð/ 5.01%, and /ŋ/ 1.68%).

### CaST procedures

Each onset and coda consonant was presented at three SNR offsets (-6, 0, +6 dB) relative to a consonant-specific baseline SNR, B. The SNRs for each syllable (B-6, B, or B+6 dB) varied randomly from trial to trial with the constraint that each onset and coda consonant was presented 12 times at each SNR, with equal sampling of four different talker voices and the three vowels. The noise level was adjusted dynamically over a pre-identified, 100-ms mid-portion of the vowel [25], so that onset and coda consonants were presented at the same relative-to-baseline SNRs. CVC levels were randomly roved from 70–75 dB SPL in 1-dB steps.

Preliminary studies revealed that OHI listeners’ consonant-identification performance was much poorer than that of ONH listeners, and varied significantly with audiometric thresholds. Therefore, baseline SNRs (B) were adjusted individually based on their OHI listeners’ audiometric thresholds. As a result, mean baseline SNRs used for testing different consonants differed in the ONH and OHI groups (see S2 Table). The maximal SNR used to present any consonant was truncated to 40 dB because, at such low noise levels most OHI listeners had difficulty in perceiving the noise at all. The goal of these SNR adjustments was to equate the overall performance of OHI listeners in aided-listening conditions with the performance of ONH listeners (d’ = 2.2).

Presentation software version 13.0 (NeuroBehavioral Systems, Albany, CA) was used for stimulus delivery, masking noise adjustment, response monitoring, and d’ calculations. Immediately before the first CaST session, listeners were briefed with written and oral instructions and received 5–15 min of training in identifying CVCs presented in quiet. In order to evaluate hearing aid benefit, each listener underwent two test sessions, one in aided and one in unaided listening conditions. The order of aided and unaided testing sessions was counterbalanced across listeners.

For listeners performing above or below criterion d’ levels of 2.20, the estimated SNRs needed to achieve criterion performance were computed using group psychometric slopes (d’/SNR), calculated separately for Groups A, B, and C consonants in the ONH group, and in the OHI group in unaided and aided conditions. In 1.9% of Group A and B consonant trials calculated SNR values were below -15 dB or above 100 dB, and were truncated to those values. In unaided OHI listeners, extrapolated SNRs over 100 dB for Group C consonants were relatively common because many OHI listeners were unable to accurately identify Group C consonants even at B+6 SNRs, and Group C psychometric slopes were very shallow (see below).

### Sentence testing materials

Speech intelligibility in noise was measured using two widely-used sentence-based tests, the HINT and QSIN. The HINT uses simple, high-context sentences spoken in an expressive manner and presented in a steady-state speech-shaped noise. The QSIN uses slightly more complex and lower-context sentences spoken in a more neutral manner and presented in multi-talker babble.

### Sentence testing procedures

Testing was performed in the sound field using the same speakers and Presentation software used for consonant testing. On each trial, the sentence and noise were played concurrently through both speakers. Sentences were presented at 70 dB SPL.

Each HINT list contained 20 sentences. The SNR was decreased in 4-dB steps from the starting SNR until the first incorrect response. Thereafter, the SNR was adjusted in 2 dB steps in a 1-up, 1-down procedure to estimate 50% correct intelligibility. The threshold was calculated by averaging the SNRs over the last 16 sentences in a block. In the ONH group, 12 HINT thresholds were measured over the course of three test sessions (four thresholds were measured at each session), without repeating sentences. For the OHI group, three HINT thresholds were measured for each listener in both aided and unaided conditions.

QSIN lists contained 6 sentences each. The starting SNR was 25 dB. The SNR decreased in 5-dB steps, regardless of response accuracy, until it reached the final SNR of 0 dB. The intelligibility threshold was estimated using the method described in the test manual, and is reported as SNR loss (the increase in SNR required relative to a baseline group of normal-hearing listeners). For the ONH group, a total of 18 threshold estimates were measured over the three sessions (six thresholds per session). For the OHI group, four thresholds were measured in both aided and unaided conditions. List orders were counterbalanced across subjects, as was the order of unaided and aided testing.

### Statistical analysis

The data were analyzed with analysis of variance for multifactorial repeated measures using the open-source CLEAVE program (T. J. Herron, http://www.ebire.org/hcnlab). The original degrees of freedom are reported for each test, with the significance levels adjusted using the Box/Greenhouse-Geisser correction for inhomogeneity of variance when appropriate [53]. Partial omega squared (ω^2^) size of effect values are also reported for all significant results. ANOVAs were supplemented with correlation and regression analyses [54] in order to explore the relationships between audiometric thresholds, consonant-identification thresholds, and SeRTs.

## Results

### Overall consonant identification accuracy

**Fig 2:**
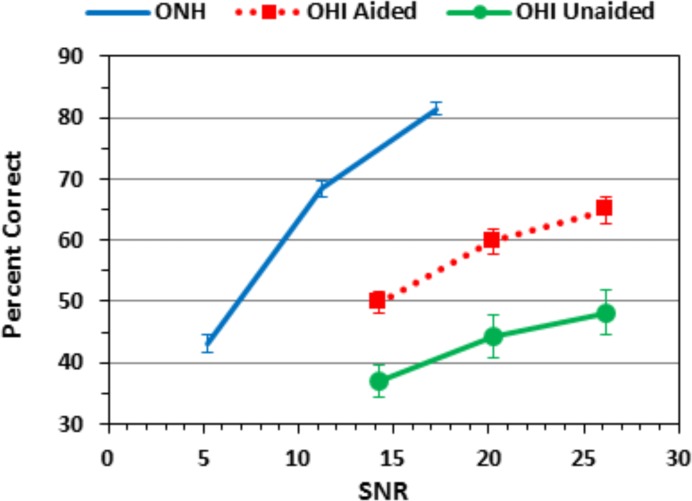
Percent correct scores for the ONH listeners (gray) and for aided (red) and unaided OHI (green) listeners averaged over all consonants in onset and coda positions. The horizontal positions of the curves were determined by the mean levels of SNRs for ONH and OHI listener groups. Error bars show standard errors.

Fig. 2 shows the average consonant-identification accuracy at different SNRs for ONH listeners, and for OHI listeners in unaided and aided conditions. Although the OHI listeners were tested at average SNRs 9.3 dB above those used to test ONH listeners, their consonant-identification performance in both aided and unaided conditions was markedly impaired. Indeed, the accuracy of unaided OHI listeners at B and B+6 dB SNRs was similar to the performance of ONH listeners at B-6 dB SNRs, an absolute SNR difference of 15–20 dB. Not surprisingly, the inter-listener threshold variability was also considerably larger in OHI than ONH listeners, as reflected in the error bars in Fig. 2.

We first analyzed overall consonant-identification accuracy using ANOVA with Hearing-loss (unaided OHI vs. ONH), Position (Onset and Coda), Vowel (/ɑ/, /i/, and /u/), and relative SNR (B-6, B, and B+6) as factors. This analysis confirmed the highly significant effect of Hearing-loss [F(1,38) = 30.1, p < 0.0001, ω^2^ = 0.43]. The main effect of Position was not significant [F(1,38) = 1.79], nor was there a significant Group x Position interaction [F(1,38) = 1.99]. This indicates that the SNR adjustments of onset and coda (coda SNRs were increased by 3–4 dB SNR) effectively equated onset and coda performance in both the ONH and OHI groups: i.e., OHI listeners did not have disproportionate deficits in coda identification. The expected large main effect of SNR was found [F(2,76) = 524.6, p < 0.0001, ω^2^ = 0.93], and there was also a Hearing-loss x SNR interaction [F(2,76) = 169.3, p < 0.0001, ω^2^ = 0.81]. This reflected the shallower psychometric slopes seen in unaided OHI listeners than in ONH listeners that will be discussed in more detail below.

The CaST also included vowel-specific SNR adjustments [25] that equated consonant-identification performance in syllables containing different vowels in young and ONH listeners [23]. Nevertheless, the ANOVA revealed a large Vowel main effect [F(2,76) = 49.0, p < 0.0001, ω^2^ = 0.55], as well as a highly significant Hearing-loss x Vowel interaction [F(2,76) = 31.0, p < 0.0001, ω^2^ = 0.43]. Separate analyses showed that consonant-identification accuracy was not influenced by Vowel in the ONH group [F(2,30) = 0.25, NS]. In contrast, consonant identification was strongly influenced by Vowel in unaided OHI listeners [F(2,46) = 54.62, p < 0.0001, ω^2^ = 0.70], with more accurate consonant identification in syllables containing /ɑ/ (48.2%) than in syllables containing /u/ (43.9%) or /i/ (37.4%). There was also a significant Hearing-loss x Position x Vowel interaction [F(2,76) = 7.29, p = 0.002, ω^2^ = 0.14] that reflected larger Vowel influences on onset than coda consonants in the OHI listeners.

### HA benefit on consonant-identification accuracy

Overall aided performance of OHI listeners is shown in Fig. 2 (dashed red line). To analyze HA benefit in OHI listeners, we first performed a repeated measures ANOVA in OHI listeners with the factors of Amplification (unaided vs. aided), Position (onset, coda), Vowel, and SNR. Amplification produced a highly significant mean 15% improvement in identification accuracy [F(1,23) = 35.2, p < 0.0001, ω^2^ = 0.60]. The Position factor reached marginal significance [F(1,23) = 5.61, p < 0.03, ω^2^ = 0.17], reflecting the fact that OHI listeners were slightly more accurate in identifying coda consonants (51.5% correct) than onset consonants (49.9% correct). This was due to the fact that coda consonants were presented to OHI listeners at an average SNR 4 dB greater than that of onset consonants. The significant Position effect suggests that the correction factor was slightly larger than it should have been, and that the true position advantage remained with the onset position (see below). There was no significant Amplification x Position interaction [F(1,23) = 0.16].

The Vowel factor remained highly significant [F(2,46) = 48.9, p < 0.0001, ω^2^ = 0.68]. OHI listeners were more accurate when identifying consonants in syllables containing /ɑ/ (54.3% correct) than in syllables containing /u/ (51.5%), or particularly /i/ (46.2%). There was also a highly significant Position x Vowel interaction [F(2,46) = 101.9, p < 0.0001, ω^2^ = 0.81]: inter-vowel differences in accuracy were larger in onset (14.9%) than coda (2.2%) positions. Finally, there was also a significant Amplification x Vowel interaction [F(2,46) = 11.8, p < 0.0001, ω^2^ = 0.32]. This reflected the fact that inter-vowel differences were reduced in aided conditions (e.g., the /ɑ/-/i/ difference was 10.8% in unaided conditions vs. 5.4% in aided conditions). However, vowel normalization was incomplete: inter-vowel differences remained significant when examined in the aided condition alone [F(2,46) = 16.94, p < 0.0001, ω^2^ = 0.41].

Increasing SNRs produced a predictable improvement in performance [F(2,46) = 139.4, p < 0.0001, ω^2^ = 0.86], with percent correct scores increasing by 13.2% as SNRs increased over the 12 dB range. There was also a significant Amplification x SNR interaction [F(2,46) = 8.22, p < 0.005, ω^2^ = 0.11], reflecting an increase in the steepness of the psychometric function with amplification, which is discussed below.

### Syllable intensity effects on consonant-identification accuracy in unaided and aided listening

In order to examine the role of audibility, we compared consonant-identification accuracy for syllables presented in the high (73–75 dB) and low (70–72 dB) range of roved syllable intensities. Syllable intensity had no significant effect on performance in ONH listeners [F(1,15) = 0.43, NS]. In contrast, unaided OHI listeners showed a significant improvement in accuracy for higher-intensity syllables [44.6% vs. 41.7%, F(1,23) = 16.59, p < 0.005, ω^2^ = 0.40]. A comparison of ONH and OHI listeners showed a significant Hearing-loss x Intensity interaction [F(1,38) = 8.03, p < 0.008, ω^2^ = 0.15].

Next we analyzed whether amplification normalized these audibility effects in an ANOVA comparing unaided vs. aided OHI listeners. This analysis again revealed a significant effect of syllable intensity [2.45%, F(1,23) = 29.18, p < 0.0001, ω^2^ = 0.55], but no interaction with Amplification [F(1,23) = 0.75, NS].

### Identification thresholds of individual consonants

**Fig 3:**
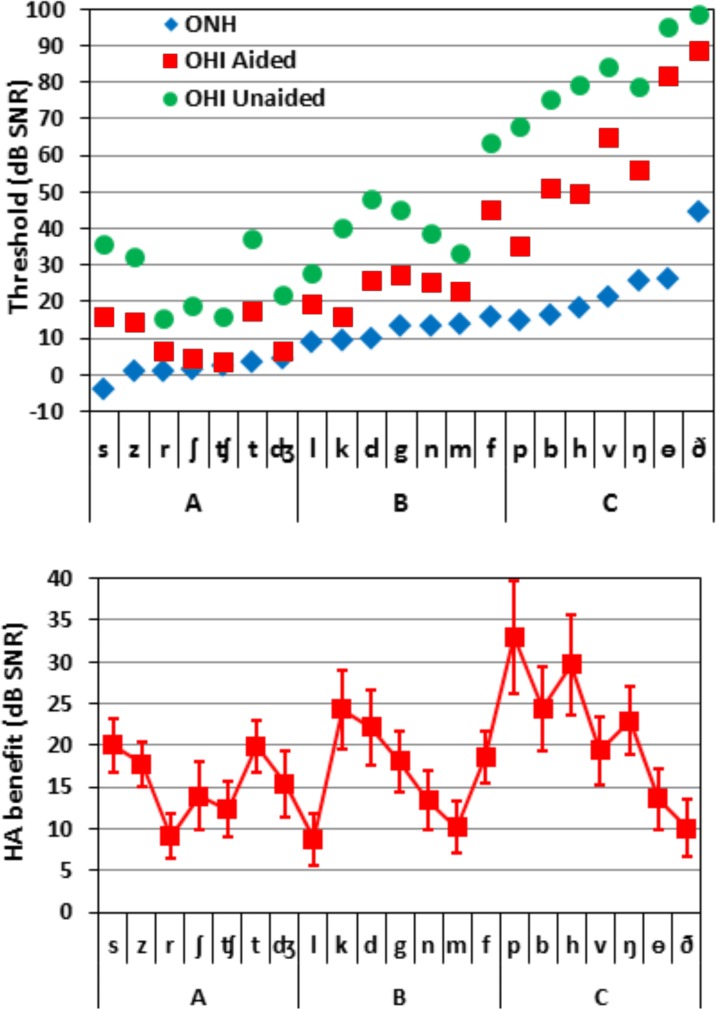
(top). Identification thresholds of the 21 consonants, averaged over onset and coda position where possible. Blue diamonds show ONH thresholds; green circles show unaided OHI thresholds; and red squares show aided OHI thresholds. The consonants on the abscissa are arranged according to increasing thresholds for normal-hearing listeners, which also determined membership in consonant Groups A, B, and C. (Bottom) HA benefit for the 21 consonants. Error bars show standard errors.

Fig. 3 (top) shows the identification thresholds for individual consonants for ONH, aided-OHI, and unaided-OHI listeners. The consonants are shown in order of increasing consonant-identification thresholds in normal-hearing listeners [41]. We first compared unaided OHI and ONH listeners with Position (onset and coda), and Consonant (all 19 consonants that appeared in both the onset and coda position, with /ŋ/ and /h/ excluded) as factors. There was a highly significant effect of Group [F(1,38) = 62.6, p < 0.0001, ω^2^ = 0.61], reflecting higher thresholds in OHI listeners (mean 47.8 dB) than ONH listeners (10.5 dB). The Position effect was also significant [F(1,38) = 25.3, p < 0.0001, ω^2^ = 0.38]: consonants in onset position required lower SNRs than coda consonants (by 2.9 dB), without a significant Hearing-loss x Position interaction [F(1,38) = 0.00, NS].

The Consonant factor was also highly significant [F(18,684) = 176.7, p < 0.0001, ω^2^ = 0.82], reflecting the fact that the identification thresholds for individual consonants varied by 50 dB among ONH listeners [23] and by over 80 dB among OHI listeners. In addition, the effect of hearing loss differed for different consonants, producing a significant Hearing-loss x Consonant interaction [F(18,684) = 33.7, p < 0.0001, ω^2^ = 0.46].

A subsequent ANOVA with Hearing-loss (ONH, unaided OHI) and Consonant-Group (A, B, C) as factors showed significant effects of Hearing-loss [F(1,38) = 66.5, p < 0.0001, ω^2^ = 0.63] and Consonant-Group [F(2,76) = 606.8, p < 0.0001, ω^2^ = 0.92] (see S1 Fig.). The Hearing-loss x Consonant-Group interaction was also highly significant [F(2,76) = 38.9, p < 0.0001, ω^2^ = 0.49], reflecting the larger threshold elevations in OHI listeners for Group C consonants (60.3 dB) than for Group A (24.8 dB) or Group B (31.3 dB) consonants. Indeed, as seen in Fig. 3 (top), the thresholds of many Group C consonants exceeded 70 dB, indicating that the performance of OHI listeners remained well below d’ = 2.2 (approximately 67% correct), even at the highest SNRs tested. When only Group A and B consonants were included in the ANOVA, the Hearing-loss x Consonant-Group interaction failed to reach significance. This was due to the fact that the effects of hearing loss were not uniform across or within these consonant groups. For example, among Group A consonants, /s/ thresholds were the lowest of all consonants in ONH listeners, but were elevated by more than 40 dB in OHI listeners.

### Audiometric factors influencing unaided consonant-identification thresholds

We analyzed the correlations between audiometric thresholds and unaided consonant-identification thresholds for the different consonant groups, mean consonant-identification thresholds, psychometric slopes, and SeRTs (discussed below). We found strong correlations between consonant-identification performance and audiometric thresholds (S3 Table). Because the high-frequency PTAs (HPTA, 3–8 kHz) were uniformly elevated in this OHI listener group, correlations were stronger for the PTA, and particularly for selected middle frequencies and the mid-frequency pure tone average (MPTA, 1–3 kHz). Despite the fact that the MPTA was highly correlated with consonant-identification performance (r = 0.86), unaided listeners with MPTAs in the middle range (35–45 dB) showed consonant-identification thresholds that varied by more than 35 dB.

We also analyzed the correlations between audiometric thresholds and unaided identification thresholds for the individual consonants (S4 Table). Overall, the 2 kHz threshold showed the greatest correlation with virtually all consonant thresholds, while the 1 kHz and 3 kHz thresholds were significantly correlated with 17 and 18 consonant thresholds, respectively. The greatest correlations of the higher frequency thresholds were with Group A sibilants, affricates, and the plosive /t/.

Table 1 provides additional details about the unaided performance of individual OHI listeners, showing their MPTA, CaST threshold elevations for different consonant groups (re: ONH thresholds), CaST z-scores (based on the mean and standard deviation of CaST thresholds in the ONH group), and psychometric function slopes (%/dB). There was a broad range of average consonant-identification thresholds (9.9 to 71.8 dB) among unaided OHI listeners, due to the fact that many OHI listeners had mean Group C consonant thresholds that exceeded 90 dB. The average z-score elevation of OHI listeners’ consonant-identification thresholds was 19.9: only one unaided OHI listener showed mean thresholds within the normal range (i.e., z-score < 2.0).

**Table 1:** Mid-frequency PTA (MPTA), unaided consonant identification threshold elevations (re: ONH listeners) for Groups A, B, and C consonants, mean consonant identification threshold elevations, and z-score elevations for OHI listeners.

Listener	MPTA	CaST Group A	CaST Group B	CaST Group C	Mean CaST	CaST z-score	CaST P/S Slope	Mean SeRT	SeRT z-score
1	39.2	8.8	12.5	51.7	24.3	12.8	1.68	3.7	3.9
2	38.3	6.9	-1.5	25.8	10.4	5.5	1.81	3.5	3.7
3	44.2	21.6	33.3	73.1	42.7	22.5	0.87	6.7	7.0
4	40.8	0.6	6.3	42.3	16.4	8.6	1.06	1.3	1.4
5	56.7	15.4	38.1	72.4	42.0	22.1	1.06	5.2	5.4
6	54.2	24.8	46.5	73.5	48.3	25.4	0.85	4.1	4.2
7	18.3	6.1	-3.3	25.6	9.5	5.0	1.94	1.2	1.3
8	42.5	30.7	35.8	71.6	46.1	24.2	0.94	9.0	9.4
9	53.3	40.4	54.9	75.8	57.0	30.0	-0.02	9.0	9.4
10	30.0	-11.2	-6.6	13.9	-1.3	-0.7	1.04	0.9	1.0
11	61.7	49.6	57.6	73.8	60.4	31.8	0.73	14.7	15.4
12	48.3	30.8	30.9	71.5	44.4	23.4	1.15	7.6	8.0
13	37.5	18.2	8.1	38.5	21.6	11.4	1.88	4.1	4.3
14	49.2	26.9	36.0	69.7	44.2	23.2	0.49	5.6	5.9
15	59.2	46.4	57.3	75.8	59.9	31.5	0.22	14.2	14.8
16	57.5	46.2	49.2	73.6	56.4	29.7	0.40	10.4	10.9
17	43.3	4.0	3.6	29.1	12.2	6.4	0.92	1.4	1.5
18	48.3	45.5	42.3	74.6	54.1	28.5	1.04	14.2	14.9
19	49.2	30.4	46.9	75.8	51.0	26.8	0.83	8.8	9.3
20	41.7	30.5	28.4	63.5	40.8	21.5	0.62	5.1	5.4
21	50.0	21.9	57.4	75.8	51.7	27.2	0.15	9.0	9.4
22	62.5	48.1	58.5	75.3	60.6	31.9	0.26	16.1	16.9
23	27.5	2.6	-5.5	20.7	5.9	3.1	1.70	1.8	1.9
24	44.2	28.3	38.4	75.8	47.5	25.0	0.85	5.2	5.5
**Mean**	**45.7**	**23.9**	**30.2**	**59.1**	**37.7**	**19.9**	**0.94**	**6.9**	**7.3**

Also included are mean psychometric slopes (P/S in %/dB SNR), and mean SeRT elevations and SeRT elevation z-scores.

Table 1 also includes the psychometric slopes for unaided OHI listeners. There was no overlap in psychometric slopes between the unaided OHI and ONH listeners: the steepest psychometric slope in the OHI group (1.94%/dB) was less than the shallowest psychometric slope of any ONH listener (2.66%/dB). Psychometric slopes in unaided OHI listeners correlated negatively with the MPTA [r = -0.74, t(21) = 5.16, p < 0.0001] and PTA [r = -0.78, t(21) = 5.85, p < 0.0001], but not the HPTA [r = -0.21, t(21) = 0.96, NS]. Mean consonant-identification thresholds were significantly lower in listeners with steeper psychometric slopes [r = -0.73, t(21) = 5.01, p < 0.0001].

Regression analysis showed a small but marginally significant correlation between CaST thresholds and age [r = 0.38, t(21) = 1.88, p < 0.04]. Multiple regression analysis, examining CaST thresholds with age and MPTA as factors, revealed that only the MPTA had a significant independent contribution [t(20) = 7.1, p < 0.0001]. This suggests that the correlation between consonant-identification thresholds and age was largely mediated by the increase in mid-frequency audiometric thresholds in older listeners.

### Hearing aid benefit on identification thresholds for individual consonants

All consonants showed significant HA benefit (S5 Table), with significance levels ranging from 0.004 for /l/, to less than 0.0001 for 12 of the consonants. Fig. 3 (bottom) shows the magnitude of HA benefit for individual consonants. An ANOVA for repeated measures with Amplification (aided vs. unaided), Consonant (the 19 that appeared in both onset and coda positions), and Position (onset and coda) showed a highly significant effect of Amplification [F(1,23) = 48.75, p < 0.0001, ω^2^ = 0.67]: aided thresholds were reduced by an average of 17.7 dB in comparison with unaided thresholds. There was also a significant effect of Consonant [F(18,414) = 243.29, p < 0.0001 ω^2^ = 0.91], reflecting the large range in consonant-identification thresholds. Finally, there was a significant effect of Position [F(1,23) = 8.54, p < 0.005 ω^2^ = 0.16], due to lower SNRs at onset than coda positions. There was no significant Amplification x Position interaction [F(1,23) = 0.63, NS], and the Amplification x Consonant x Position interaction did not reach significance [F(18,414) = 1.79, NS].

However, there was a significant Amplification x Consonant interaction [F(18,414) = 5.28, p < 0.001, ω^2^ = 0.08], indicating that HA benefit differed for different consonants, ranging from 8.8 dB SNR for /l/, to 33 dB SNR for /p/. Mean HA-benefit was slightly smaller for Group A (15.5 dB) and Group B (16.5 dB) consonants than for Group C consonants (21.5 dB) [F(2,46) = 3.76, p < 0.05, ω^2^ = 0.10]. However, these benefits were superimposed on much larger Group C threshold elevations in unaided listeners. As a result, HAs remediated 65% of the Group A deficit, 55% of the Group B deficit, and 36% of the Group C deficit, relative to the thresholds in ONH listeners.

**Fig 4:**
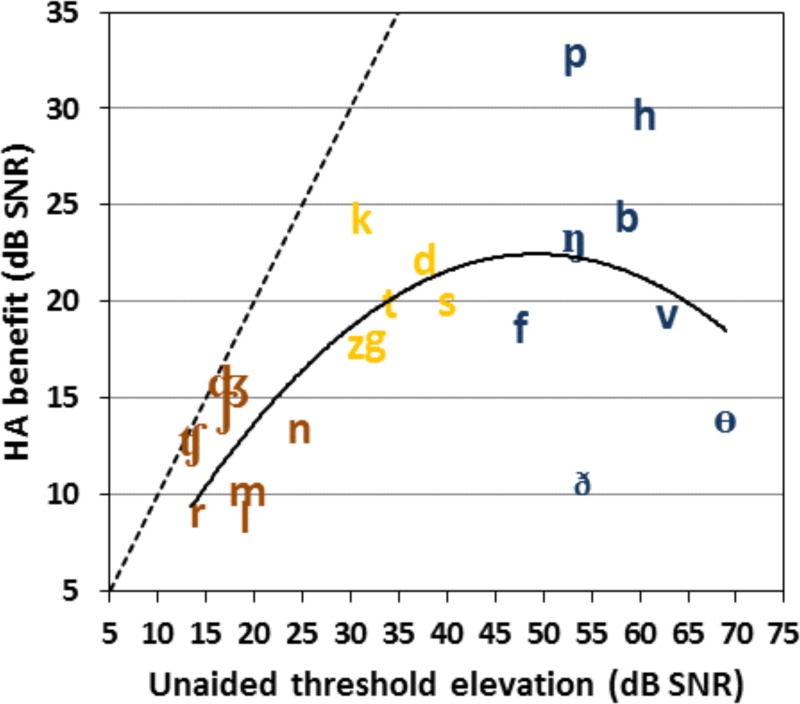
HA benefit plotted as a function of unaided threshold elevation. The dotted line illustrates the case where the HA benefit equals the unaided threshold elevation, i.e., where aided-OHI and ONH consonant identification would be the same. The solid curve is the second-order polynomial fit for these data. The consonants are color-coded according to their unaided threshold elevations (brown for under 30 dB, yellow for 30–45 dB and dark blue for over 45 dB). See text for further discussion.

Fig. 4 shows the relationship between unaided thresholds and HA benefit for different consonants. If HAs completely restored normal thresholds, HA benefit for different consonants would fall on the straight diagonal line. A low threshold-elevation (<25 dB), low HA-benefit (<16 dB) brown cluster included liquids, nasals, affricates, and the lower-frequency sibilant /ʃ/ from Groups A and B. These consonants were relatively well identified in unaided listening conditions, limiting potential HA benefit. A medium threshold-elevation (30–40 dB), medium HA-benefit (17–25 dB) yellow cluster included most plosives and higher-frequency sibilants, also from Groups A and B. The remaining consonants in the high-threshold-elevation (>45 dB) blue cluster included the non-sibilant fricatives, the front plosives /p/ and /b/, /h/, and the hard-to-identify nasal /ŋ/. The blue cluster includes a much broader range of HA benefit than the brown and yellow clusters.

In order to evaluate the degree to which HAs restored consonant identification to normal levels, we performed another ANOVA comparing ONH vs. aided OHI listeners with Position (onset, coda) and Consonant (19 consonants that occurred in both positions) as factors. The expected main effects were found for Group [F(1,38) = 44.77, p < 0.0001 ω^2^ = 0.53], Position [F(1,38) = 18.73, p < 0.0001 ω^2^ = 0.31], and Consonant [F(18,684) = 370.36, p < 0.0001 ω^2^ = 0.90]. ONH listeners had better thresholds than aided OHI listeners (10.5 versus 30.6 dB SNR); there was an onset advantage (21.4 versus 23.6 dB SNR), and consonant thresholds ranged from 2.5 (/ʧ/) to 70.7 dB (/ð/). A significant Consonant x Position interaction was also found [F(18,684) = 38.62, p < 0.0001 ω^2^ = 0.49], reflecting the fact that some consonants had onset advantages (e.g., /d/, /g/, /m/, /n/, /s/, and /t/) while others had coda advantages (e.g., /b/, /ʧ/, /l/, /r/, and /ʃ/). A significant Hearing-loss x Consonant interaction was also observed [F(18,684) = 56.93, p < 0.0001 ω^2^ = 0.31], reflecting the non-uniform elevations in aided thresholds shown in Fig. 3.

While the overall magnitude of improvement (in dB) was smaller for consonants with better unaided thresholds, the relative normalization of hearing was, in general, larger for these consonants (S2 Fig.). For example, when using their HAs, more than 40% of OHI listeners had affricate, /ʃ/, and /r/ thresholds within the normal range, and more than 25% had thresholds within the normal range for /l/, /k/, /g/, /m/, /n/, and /p/. However, performance was poorly normalized for other Group A consonants, including several that occur frequently in conversational speech (i.e., /s/, /z/, and /t/).

### Types of phonetic errors in unaided and aided listening

**Fig 5:**
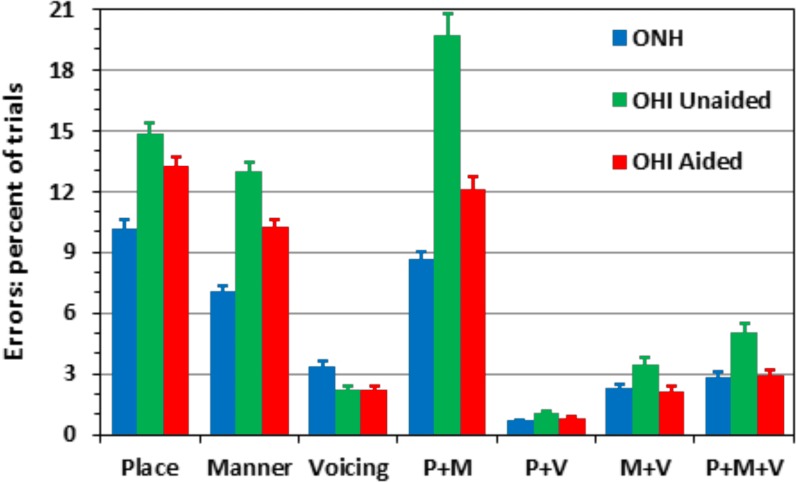
Incidence of different types of phonetic errors for ONH listeners, and for unaided (green) and aided (red) OHI listeners. Error bars show standard errors.

Fig. 5 shows the percentage of trials with different types of consonant feature errors for ONH listeners (blue bars) and OHI listeners in unaided (green) and aided (red) conditions. In this analysis we used six manners (liquids, nasals, affricates, plosives, non-sibilant fricatives, and sibilants) and three places of articulation (front, middle, back). In unaided conditions, OHI listeners showed greater overall error rates (59%) than ONH listeners (35%), and also showed a different pattern of phonetic errors. In particular, OHI listeners made *fewer* Voicing (V) errors (2.2% vs. 3.3% of trials), likely reflecting the higher SNRs used for OHI testing. All other types of errors were markedly increased in OHI listeners, including Place (P) errors (+ 4.7%), Manner (M) errors (+5.9%), and particularly, Place + Manner (P+M) errors (+11.1%). Overall errors that included Manner nearly doubled (41.2% versus 20.9%), and those that included Place showed almost as great an increase (40.7% versus 22.4%). Clearly, the OHI deficit in consonant perception reflected inordinate difficulties in discriminating the Manner and Place of articulation.

Errors in aided listening conditions are shown in the red bars in Fig. 5. Hearing aids reduced the overall number of errors by 26% and altered the relative incidence of errors of different types. HAs did not reduce Voicing errors [F(1,23) = 0.23], but significantly reduced Place errors [F(1,23) = 10.31, p < 0.005, ω^2^ = 0.29] and produced even larger reductions in Manner errors [F(1,23) = 38.81, p < 0.0001 ω^2^ = 0.59] and Place + Manner errors [F(1,23) = 21.64, p = 0.0001 ω^2^ = 0.47]. Although the remaining combined multi-feature errors occurred on less than 10% of trials, all three also showed relatively large HA-related reductions: P+V, [F(1,23) = 9.39, p < 0.006, ω^2^ = 0.27]; M+V [F(1,23) = 33.87, p < 0.0001 ω^2^ = 0.59]; and P+M+V [F(1,23) = 22.97, p < 0.0001, ω^2^ = 0.49]. Such reductions in P+M and other multi-feature errors indicate that the HAs improved the consonant information available, even when aided listeners were unable to fully identify the consonant. Similarly, the shift toward a preponderance of place errors indicates performance normalization because place errors predominated in ONH listeners.

### Consonant confusions in unaided and aided listening

**Fig 6:**
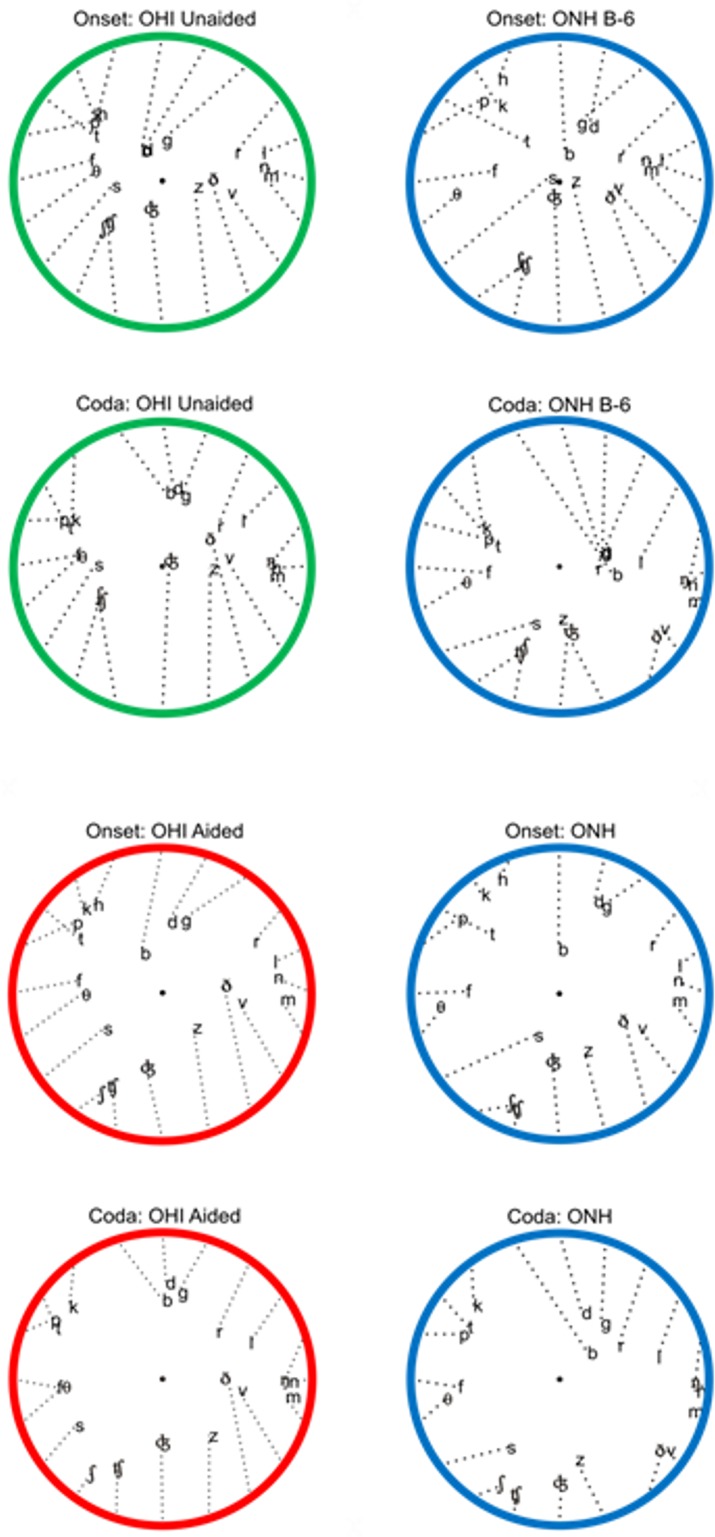
Barycentric plots for unaided (green) and aided (red) OHI listeners, and for ONH listeners (blue) in SNR conditions producing similar overall consonant identification performance.

The consonant confusion matrices are included in S6A-B Table (unaided OHI listeners) and S7A-B Table (aided OHI listeners). Fig. 6 shows the consonant confusions for onset and coda consonants, visualized with barycentric displays for ONH listeners and OHI listeners in unaided (green) and aided (red) conditions. Consonants were initially placed in equidistant positions around a unit circle based on Voicing, Manner, and Place features using an optimized *a priori* consonant ordering. The location of each consonant was then modified as an average of its initial position weighted by its hit rate and the position of every other consonant weighted by its false response probability when the consonant was presented. Two movement iterations were used to generate the cluster plots shown, displacing each consonant from its initial position towards the locations of the consonants with which it was confused (dotted lines in Fig. 6).

The confusions of unaided OHI listeners (top left) and ONH listeners at B-6 SNRs (blue, top right) were obtained at similar overall performance levels (43% correct). However, there were differences in the patterns of confusions in the two groups of listeners. For example, in unaided OHI listeners, the unvoiced onset plosives /p/, /t/, and /k/, as well as /h/ (Fig. 6, row 1), were virtually indistinguishable from each other, forming a superimposed cluster in the upper left quadrant of the confusion circle. A relatively high frequency of confusions of these consonants with unvoiced fricatives and /s/ resulted in the movement of this consonant cluster toward the unvoiced fricative clusterl. For ONH listeners, these consonants were less frequently confused with each other (i.e., showed less superposition), even at the most difficult SNRs, while /t/ was confused with a number of other consonants, displacing it toward the center of the circle.

Amplification (Fig. 6, rows 3 and 4) moved all consonants away from the circle center, reflecting a reduction in Manner and multi-feature errors. In addition, the consonants that differed only in Place moved further apart from each other (e.g., /p/, /t/, and /k/; /b/, /d/, and /g/; /m/, and /n/; /s/ and /ʃ/), indicating a reduction in place errors. However, a number of consonants, including the fricatives /f/, /θ/, /ð/, and /v/, remained far from the circle edge, indicating abnormally high manner errors. The relatively tight clustering of the sibilants and affricates at the bottom of the ONH circles also differed in aided OHI and ONH listeners (/s/-/z/ and /ʧ/-/ʤ/) due to the reduction of Voicing errors in OHI listeners.

### Psychometric function slopes in unaided and aided listening

We compared the slopes of the psychometric functions for different consonants in unaided and aided listening conditions with the slopes of ONH listeners (S3 Fig.). OHI listeners showed markedly reduced slopes in both unaided (a mean of 25% of the slope of ONH listeners) and aided (39% of the ONH slope) conditions. Although an ANOVA showed that HAs increased psychometric slopes [F(1,39) = 37.1, p < 0.0001 ω^2^ = 0.48], the slopes of aided OHI listeners remained much shallower than those of ONH listeners [F(1,39) = 241.8, p < 0.0001 ω^2^ = 0.86].

### Audiometric factors influencing HA benefit on consonant-identification thresholds

The MPTA explained 64% of the variance in HA benefit (r = 0.80, S4 Fig.). However, OHI listeners with intermediate MPTA thresholds (35–45 dB) showed substantial variation in HA benefit (i.e., -5 to 26 dB). Mean HA benefit also correlated strongly with unaided consonant-identification thresholds [r = 0.81, t(22) = 6.48, p < 0.0001], i.e., individuals with greater threshold elevations showed greater HA benefit. Multiple regression analysis with MPTA and unaided consonant thresholds as factors accounted for 70% of HA benefit variance. MPTA and unaided thresholds were highly correlated [r = 0.86, t(22) = 7.91, p < 0.0001], and neither factor made a significant independent contribution to HA benefit in the multiple regression analysis [respectively t(21) = 1.79 and t(21) = 1.89, both p < 0.10], nor did Age correlate with HA benefit [r = 0.14, NS]. Further analysis revealed that aided performance showed stronger correlations with high-frequency thresholds (S8 Table) than did unaided performance (S5 Table).

Table 2 shows the HA benefit for individual OHI listeners and includes the MPTA, HA benefit for Groups A, B, and C consonants, mean overall HA benefit, and HA benefit z-scores for improvement in consonant thresholds. HAs produced large improvements in consonant-identification thresholds (mean 17.7 dB), with a mean z-score benefit of 8.2. However, HA benefit on consonant identification varied substantially among listeners, ranging from -5.2 dB (better performance in unaided conditions) to 41.2 dB. Overall, 83% of OHI listeners showed significant HA benefit on consonant-identification thresholds (i.e., z-score improvements > 2.0).

**Table 2:** Mid-frequency PTA and HA benefit on consonant identification measures for different consonant groups for individual listeners.

		**CaST HA benefit**					**SeRT HA benefit**	
Listener	MPTA	Group A	Group B	Group C	Mean	Z-score	SeRT	Z-score
1	39.2	7.4	14.7	35.8	19.3	9.6	0.8	0.7
2	38.3	1.4	-1.9	15.6	5.0	2.5	-0.5	-0.4
3	44.2	13.8	14.5	26.9	18.4	7.8	1.8	1.5
4	40.8	4.9	8.4	36.5	16.6	7.7	0.2	0.2
5	56.7	10.4	20.9	32.3	21.2	9.3	1.1	0.9
6	54.2	20.8	37.6	42.0	33.5	15.4	1.1	1.0
7	18.3	5.3	0.3	4.6	3.4	1.9	0.1	0.1
8	42.5	21.3	21.2	34.6	25.7	11.8	4.0	3.4
9	53.3	32.9	31.7	21.3	28.7	13.6	4.5	3.8
10	30.0	-7.9	-6.0	-1.7	-5.2	-3.1	-1.1	-0.9
11	61.7	37.5	33.0	20.3	30.2	14.6	9.5	8.2
12	48.3	22.1	13.4	27.1	20.8	9.4	3.9	3.4
13	37.5	0.7	-7.7	-7.9	-5.0	-2.6	-0.2	-0.2
14	49.2	16.4	17.9	25.6	20.0	9.4	0.0	0.0
15	59.2	38.5	45.1	40.0	41.2	19.3	7.8	6.7
16	57.5	28.0	17.3	16.2	20.5	9.2	2.8	2.4
17	43.3	2.3	4.4	20.7	9.2	4.8	-2.1	-1.8
18	48.3	27.7	18.6	13.7	20.0	9.6	5.6	4.8
19	49.2	7.5	16.5	14.0	12.7	5.2	-2.8	-2.4
20	41.7	10.0	1.2	3.6	4.9	2.7	2.5	2.2
21	50.0	6.1	31.4	17.5	18.4	7.8	0.3	0.3
22	62.5	41.9	40.5	32.5	38.3	17.8	10.9	9.4
23	27.5	2.6	-2.3	9.1	3.1	1.4	-0.8	-0.7
24	44.2	20.6	25.6	26.3	24.2	11.3	-1.5	-1.3
**MEAN**	**45.7**	**15.5**	**16.5**	**21.1**	**17.7**	**8.2**	**2.0**	**1.7**

Mean benefit, z-scores, and HA benefit on SeRTs in dB and z-scores are also provided.

There were strong correlations between pure-tone audiometric thresholds and HA benefit on consonant identification (S9 Table). Listeners with greater elevations in the MPTA and PTA showed greater overall HA benefit [r = 0.79, t(22) = 6.04, p < 0.0001, and r = 0.75, t(22) = 5.32, p < 0.0001, respectively]. In contrast, the HPTA (3–8 kHz) showed a significant correlation with HA benefit on Group A consonants [r = 0.42, t(22) = 2.17, p < 0.03, one-tailed], but not for other consonant groups or with overall consonant identification benefit. We found no difference in the benefits of HAs with and without non-linear frequency compression [r = 0.28, t(22) = 1.37, p < 0.20].

### SeRTs in unaided conditions

Unaided OHI listeners produced mean HINT SeRTs of 4.1 dB, and mean QSIN SeRTs of 8.0 dB (S5 Fig.). Average SeRT thresholds for unaided OHI listeners fell well below their mean identification thresholds for any consonant. Indeed, mean consonant-identification threshold elevations in unaided conditions (37.7 dB) were more than 30 dB above mean SeRT elevations (6.9 dB).

SeRTs were first analyzed with an ANOVA with Hearing-loss (ONH, unaided OHI) and Test (QSIN, HINT) as factors. This showed a significant Hearing-loss main effect [F(1,38) = 33.8, p < 0.0001 ω^2^ = 0.41], due to an average SeRT elevation of 6.6 dB in the OHI listeners (7.7 for the QSIN and 5.9 for the HINT). Test was also a significant factor [F(1,38) = 63.9, p < 0.0001, ω^2^ = 0.53] because QSIN SeRTs showed greater elevations than HINT SeRTs. Finally, there was a Hearing-loss x Test interaction [F(1, 38) = 4.1, p = 0.05, ω^2^ = 0.09], reflecting greater OHI threshold elevations on the QSIN than on the HINT.

Normative SNRs for the HINT and QSIN were established using ONH data [23] to set upper SeRT limits for normal performance. Table 1 (above) shows that the majority of unaided OHI listeners had SeRTs outside the normal range, with an average z-score of 7.3. However, 21% of OHI listeners had mean SeRTs within the normal range, including 38% who fell within the normal range for the HINT, and 26% who fell within the normal range on the QSIN.

### Factors predicting SeRTs in OHI listeners

SeRT variability was substantially increased in the OHI group relative to ONH listeners. Individual HINT SeRTs ranged from -1.8 to 10.9 dB among OHI listeners, and QSIN SeRTs ranged from 0 to 20.0 dB. OHI listeners with better audiometric thresholds had better SeRTs, with the highest correlations seen for the MPTA [r = 0.77, t(22) = 5.66, p < 0.0001] and the 2 kHz threshold [r = 0.81, t(22) = 6.48, p < 0.0001]. Audiometric correlations for the HINT and QSIN showed patterns similar to those seen for consonant-identification thresholds.

**Fig 7:**
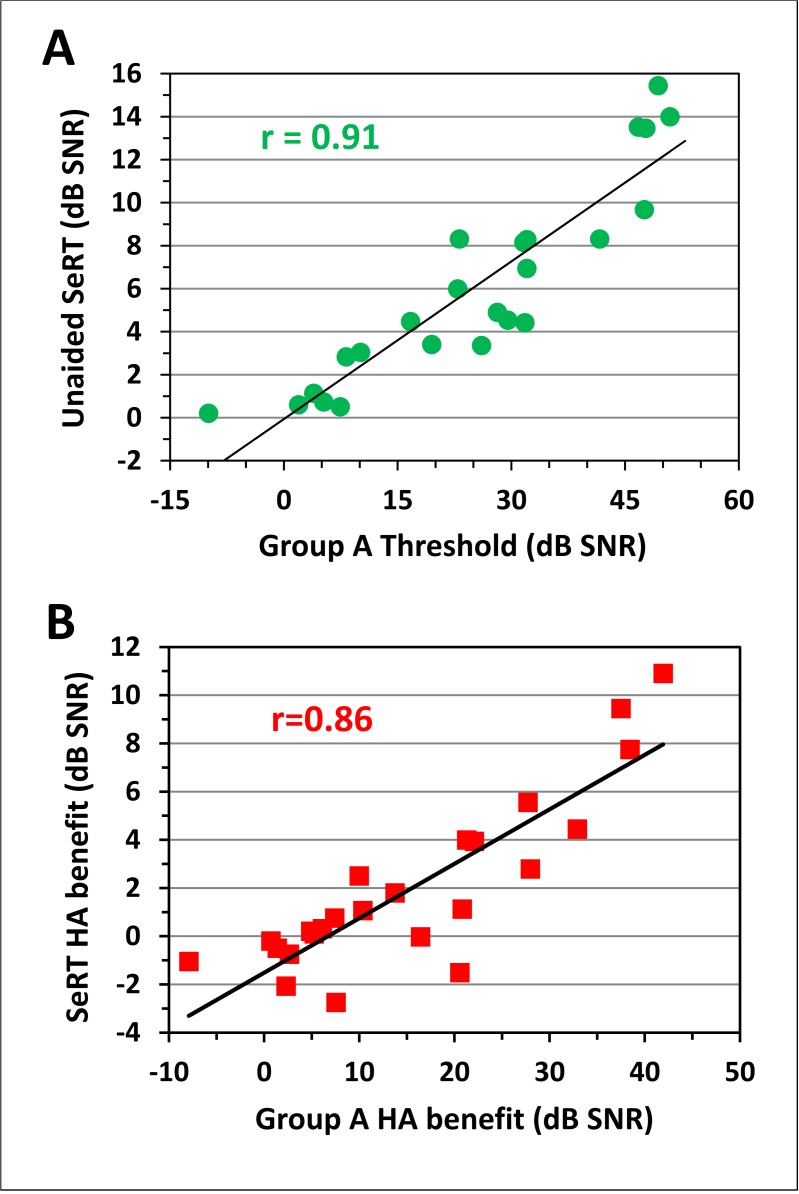
Scatter plots showing the relation between listeners’ SeRTs and unaided Group A consonant identification thresholds (Panel A) and the relation between HA benefit on Group A consonants and HA benefit on SeRTs (Panel B).

SeRT performance in unaided listening conditions was strongly correlated with consonant-identification thresholds [r = 0.86, t(22) = 7.91, p < 0.0001]. Fig. 7 (top) shows that the greatest correlation was between Group A consonant thresholds and SeRTs [r = 0.91, t(22) = 10.3, p < 0.0001], exceeding the correlations with Group B [r = 0.84, t(22) = 7.3, p < 0.0001] or Group C [r = 0.74, t(22) = 5.2, p < 0.001] thresholds. Unlike consonant-identification thresholds, SeRTs were not significantly correlated with age [r = 0.16, NS]. Multiple regression analysis of CaST consonant-group thresholds and SeRTs showed that Group A consonant thresholds accounted for SeRT performance variations [t(20) = 3.18, p < 0.005], and that neither Group B nor Group C thresholds made significant independent contributions to SeRTs [t(20) = 2.06, p < 0.06 and t(20) = -1.59, p < 0.15, respectively]. Further multiple regression analysis including the MPTA showed that CaST Group A thresholds and the MPTA accounted for 80% of SeRT variance, and revealed a strong independent effect of CaST Group A thresholds [t(21) = 9.07, p < 0.0001], without a significant additional contribution from the MPTA [t(21) = 1.76, p < 0.15].

### HA benefit on SeRTs

A repeated-measures ANOVA was performed with Amplification (aided vs. unaided) and Test (QSIN and HINT) as factors. The Amplification factor was significant due to a mean HA benefit of 2.0 dB [4.1 vs. 6.1 dB, F(1,23) = 7.52, p < 0.02 ω^2^ = 0.22]. The Test factor was also significant due to greater thresholds on the QSIN than the HINT [6.5 vs. 3.7 dB, F(1,23) = 38.42, p < 0.0001, ω^2^ = 0.62]. Finally, there was a significant Amplification x Test interaction [F(1,23) = 10.28, p < 0.005 ω^2^ = 0.29], reflecting greater HA benefit on the QSIN (3.1 dB) than on the HINT (0.9 dB). Separate ANOVAs revealed significant HA benefit on the QSIN [F(1,23) = 10.05, p < 0.005, ω^2^ = 0.28], but not on the HINT [F(1,23) = 2.46, p < 0.15, NS].

As seen in Table 2, only 38% of OHI listeners showed significant HA benefit on SeRTs (i.e., z-score improvements > 2.0). Several audiometric factors correlated with SeRT HA benefit, including the MPTA [r = 0.62, t(22) = 3.71, p < 0.001], PTA [r = 0.43, t(22) = 2.23, p < 0.02], and HPTA [r = 0.38, t(22) = 1.90, p < 0.05].

Fig. 7 (bottom) shows the strong correlation between HA benefit on Group A consonant-identification thresholds and HA benefit on SeRTs [r = 0.86, t(22) = 8.00, p < 0.0001]. SeRT benefit was also correlated with Group B [r = 0.63, t(22) = 3.81, p < 0.0005], but not Group C benefit [r = 0.34, t(22) = 1.70, p < 0.06]. Multiple regression analysis of HA benefit on SeRTs using HA benefit for all three consonant groups showed a significant independent influence of HA benefit on Group A consonants [t(20) = 5.36, p < 0.0001], without significant additional influences of HA benefit on Group B [t(20) = -0.79, NS] or Group C [t(20) = -0.34, NS] consonants. Multiple regression analysis of HA benefit on SeRTs with the factors MPTA and HA benefit on Group A consonants revealed that HA benefit on Group A consonant thresholds had a significant influence [t(21) = 3.63, p <0.002], while the indpendent effect of MPTA fell to insignficance [t(21) = -0.32, NS].

## Discussion

We found that consonant-identification thresholds were sensitive measures of the speech discrimination deficits of unaided OHI listeners with more than 95% showing significant consonant-threshold elevations. The mean consonant-identification threshold elevations in OHI listeners in our study (37.7 dB using individual consonant thresholds) were larger than the threshold elevations of unaided OHI listeners in the prior study of Phatak et al. [10]. However, because the psychometric functions of OHI listeners were substantially shallower than those of ONH listeners [10,55], the magnitude of apparent threshold elevation would depend on the performance level used for comparison. We attempted to equate consonant-identification performance at a high level of accuracy (d’ = 2.20, approximately 67% correct). Had we estimated thresholds at 50% correct, as did Phatak et al. [10], our OHI listeners would have shown threshold elevations more similar to theirs and others [16], i.e., about 20 dB (see Fig. 2).

Our unaided OHI listeners also showed much greater impairment than those of Ahlstrom et al. [26], who measured identification performance for 19 onset and 18 coda consonants in 12 OHI listeners with slightly milder sloping hearing losses. They reported that unaided OHI listeners performed at 60% correct at the single SNR tested (+4 dB). Our OHI listeners would require SNRs in excess of 35 dB to achieve similar performance (Fig. 2). Our OHI listeners were more similar to those studied by Phatak et al. [10] and others [15] who found that the consonant-identification accuracy of many OHI listeners is less than 60%, even in quiet.

How can we explain the large differences between our results and those of Ahlstrom et al. [26]? There are a number of possible contributing factors: (1) Differences in the severity of hearing loss. On average, our listeners had slightly greater high-frequency hearing loss than listeners in Ahlstrom et al. [26]. (2) Differences in listening experience. All of our listeners wore HAs on a daily basis, whereas 83% of the listeners in Ahlstrom et al. [26] did not use HAs. HA use may result in reductions in performance in unaided listening conditions [56]. (3) Differences in SNR measurement. We adjusted noise-levels based on peak vowel intensity, whereas Ahlstrom et al. [26] adjusted noise levels based on the entire syllable, potentially increasing their overall SNRs by several dB. (4) Differences in syllable structure. We used 160 CVCs to measure each consonant threshold, while Ahlstrom et al. [26] used just two CV or VC tokens. Our larger selection and more variable token set may have increased consonant-identification difficulty. (5) Differences in consonant sets. Our tests included several consonants with high SNR thresholds (e.g., /ð/ and /h/) that were not tested by Ahlstrom et al. [26]. (6) Variable vs. fixed SNRs. We presented consonants over a range of SNRs, while Ahlstrom et al. [26] used a single, fixed SNR. A fixed SNR may permit listeners to use consonant-identification difficulty itself as a cue. For example, if no consonant is heard clearly, the listener might deduce that the syllable likely contained a hard-to-hear consonant (e.g., a non-sibilant fricative). Such a strategy would have been ineffective in our experiment because consonant SNRs were adjusted to equate the identifiability of different consonants, and each consonant was presented at multiple SNRs.

### HA benefit on consonant identification

We found that HAs produced large and highly significant improvements in the identification thresholds of all consonants. HA benefit was equivalent for onset and coda consonants and for voiced and unvoiced consonants. However, benefits varied as a function of vowel nuclei: HAs improved consonant identification more in syllables with /i/ than in syllables with /ɑ/ or /u/, partially normalizing the abnormally large inter-vowel differences seen in unaided listeners.

Our results again contrast with those of Ahlstrom et al. [26], who found no significant HA benefit on consonant identification. There are again several possible reasons for this discrepancy: (1) Fixed vs. consonant-specific SNRs. Ahlstrom et al. [26] presented all consonants at a single, fixed SNR. Because of the large range of consonant-identification thresholds in OHI listeners, at a fixed SNR some consonants would be accurately identified in both unaided and aided conditions (ceiling effect), whereas others would not be identified in either condition (floor effect). In contrast, we used consonant-specific SNR ranges so that floor and ceiling effects would not mask potential HA benefit. (2) Differences in HA design. The Oticon EPOQ HAs used by Ahlstrom et al. [26] incorporate a "Speech Guard" feature that tries to maintain speech waveform shape by reducing differential amplification across frequency channels when speech is present. To the extent that Speech Guard succeeds, it would limit the amplification of low-intensity, high-frequency consonant cues. In contrast, the Phonak multichannel compression HAs we used provided independent amplification to different frequency bands. In addition, half of our Phonak HAs also included some frequency transposition, where high-frequency cues would be transposed to lower frequencies where audiological sensitivity was increased. (3). Different experience with amplification. Only two of the listeners in Ahlstrom et al. [26] wore HAs, and neither were experienced with the Oticon EPOQ HAs used in their experiment. In contrast, all of our listeners had extensive experience with their HAs and would therefore be expected to show enhanced HA benefit due to acclimatization [57].

Although HAs reduced variability among OHI listeners, their performance remained more variable than that of ONH listeners [58,59]. For example, listeners with MPTAs of 35–45 dB showed aided thresholds that varied by more than 30 dB. Plomp [60] highlighted other abnormalities in OHI listeners, including reduced dynamic intensity range, abnormal growth of loudness, reduced temporal resolution, reduced frequency resolution, and reduced spatial localization acuity. Although multichannel compression HAs provide some compensation for reduced dynamic range and abnormal growth of loudness, they provide little benefit for other distortion losses. Because OHI listeners show varying magnitudes of distortion loss, they will show greater variability than the ONH listeners, even in aided conditions.

### Syllable intensity effects on consonant identification

We tested listeners at intensities that characterize loud conversational speech (70–75 dB SPL), whereas some other studies have used more intense stimuli [4,10,16]. Nevertheless, we found that OHI listeners continued to identify consonants more accurately in more intense syllables in aided listening conditions. This suggests that the HA gains, although well-suited to patient satisfaction, were insufficient to fully restore the audibility of less intense, high-frequency consonant cues.

### Differences in the identification of individual consonants

HA benefit on individual consonants varied as a function of the intensity and frequency of the acoustic cues needed for their identification. Unaided deficits were relatively small for consonants whose identification is based on lower frequency cues (e.g., /ʧ/, /r/, /ʃ/, and /ʤ/), and aided thresholds returned to near-normal levels, consistent with the substantial amplification of 2–4 kHz frequencies. However, in accord with previous studies[10,12,15], unaided OHI listeners exhibited greater difficulties in identifying consonants that depend on high-frequency spectrotemporal cues [4,5,61,62], including stop consonants [63], sibilants [64], and non-sibilant fricatives. The identification of these consonants was only partially normalized by HAs. For example, the unaided deficits for /s/, /z/ and /t/ were in excess of 30 dB SNR, while the aided deficits exceeded 15 dB. One likely reason is that the average pure-tone thresholds of our OHI listeners exceeded 70 dB HL at 4 kHz and above, and the HAs provided only limited amplification at these higher frequencies. In contrast, the consonants /b/, /p/, and /h/ showed large HA benefit. The relatively low-frequency /p/-/b/-/h/ cues (2–3 kHz) were well amplified by the HAs so that OHI listeners’ consonant-identification thresholds for /p/, /b/, and /h/ improved substantially in aided listening conditions.

### Phonetic errors and consonant confusions

The types of phonetic errors differed in OHI and ONH listeners. OHI listeners produced fewer Voicing errors than ONH listeners in both unaided and aided listening conditions. This likely reflected the relatively well-preserved low-frequency hearing in OHI listeners, as well as the increased SNRs used for OHI testing. However, as noted in previous studies [4,18], OHI listeners showed increased Manner and Place errors. And, in contrast to ONH listeners, where Place errors were most common, unaided OHI listeners showed predominantly Manner + Place errors. Because Place errors involve a smaller set of consonants in comparison with Manner + Place errors, this indicates that unaided OHI listeners extracted less information about consonant identity than ONH listeners, even on incorrect trials. HAs reduced both Manner and Place errors, and partially normalized the error pattern, with Place errors becoming the most common, followed by Manner + Place, and Manner errors.

As seen in the barycentric plots (Fig. 6), the increase in multi-feature consonant confusions in OHI listeners produced abnormal consonant clustering that affected some consonants (e.g., unvoiced plosives, fricatives, and sibilants) more than others (e.g., nasals and liquids). The changes in the confusion clusters that include voiced and unvoiced sibilants and affricates may be particularly important because five of the seven most-easily-identified (Group A) consonants are in this cluster. These consonants were relatively well discriminated in ONH listeners, even at low SNRs (see right column of Fig. 6). In contrast, unaided OHI listeners confused these consonants with Group C fricatives with the same voicing (see middle column of Fig. 6). Such unusual confusions, especially involving Group A consonants [41], would interfere with sentence perception in high levels of masking noise (see below).

### Psychometric functions in unaided and aided listening

OHI listeners showed shallow psychometric slopes in both unaided and aided listening conditions, as would be expected from their inaccurate consonant-identification performance, even in quiet [19,58,65]. Other studies have found that unaided OHI listeners have shallow psychometric slopes both in consonant-identification tasks [10,15] and sentence testing [66]. Part of the slope reduction may reflect vowel effects: i.e., consonants in syllables with the vowel /i/ had increased thresholds relative to syllables containing other vowel nuclei. Thus, while consonant information in syllables with different vowel nuclei emerged at similar SNRs for ONH listeners, syllables containing /i/ would require an approximate 6 dB SNR boost to be as intelligible as syllables containing /ɑ/ for OHI listeners.

Another component of the slope reduction may be the inaudibility of consonant cues, even in aided conditions. In addition, impairments in frequency resolution [67] and temporal processing [68] may flatten psychometric functions even at audible intensities [69] by increasing internal noise regardless of the SNR. In addition, OHI listeners may use suboptimal phonetic cues to identify phonemes: for example, discriminating fricatives based on frication-duration rather than frequency [64,70]. Because suboptimal cues are less reliable than normal phonetic cues, they would also contribute to shallower psychometric functions.

### SeRTs in unaided and aided listening conditions

The results of the sentence testing in OHI listeners revealed significant SeRT elevations relative to the ONH group. The QSIN elevations that we found in unaided OHI listeners were similar to those reported by Phatak [10], while the HINT threshold elevations were similar to those reported by Wilson et al. [28], but somewhat greater than those reported in comparable conditions by Ahlstrom et al. [52] and Mendel [6]. In our OHI group, 38% of HINT and 26% of QSIN SeRTs fell within the normal range of ONH listeners.

As in Phatak [10], we found much larger elevations in consonant-identification thresholds than in SeRTs (38.8 vs 5.2 dB), so that all consonants had mean identification thresolds above those of HINT and QSIN SeRTs, particularly in unaided listening conditions. These results suggest that OHI listeners would detect only a subset of consonant cues (e.g., voicing and inexact manner and place cues) at the SNRs used for SeRT testing. Thus, when processing sentences in noise OHI listeners would depend more on vowel and intonation cues [15], as well as on cognitively demanding, top-down semantic processing [15,71]. Hence, our results are consistent with many studies reporting that OHI listeners find listening to speech in noisy situations more effortful than normal-hearing listeners [72–76].

### Factors influencing SeRTs in unaided and aided listening

As in previous studies, we observed small but significant HA benefit on QSIN thresholds, but not on HINT thresholds [6,52,77,78]. The greater benefit on the QSIN may be due to increased audibility of words presented during the amplitude dips in the masking speech babble, thus more closely approximating the larger HA benefits found when sentences are presented in quiet [6].

Multiple regression analysis revealed that SeRTs in unaided OHI listeners were strongly correlated with CaST Group A identification thresholds, accounting for 83% of SeRT variance. Stronger correlations with Group A than Group B or C consonants were not surprising because Group A consonants are most easily identified at the low SNRs used in SeRT testing. Although SeRTs were also significantly correlated with the MPTA, this effect disappeared in a multiple regression analysis that included Group A consonant thresholds. Thus, audiometric influences on SeRTs appeared to be largely mediated by their influence on the identification of easily-identified consonants.

As in previous studies, HA benefit on SeRTs was greater in listeners with more hearing loss [77], particularly in the mid-frequencies (1–3 kHz). However, multiple regression analysis again revealed that the influence of mid-frequency thresholds on HA benefit disappeared when HA benefit on Group A consonants was included in the regression. In other words, HA benefit on sentence processing was mediated through HA benefit on the easiest to identify consonants.

### The relationship between consonant identification and sentence processing

Unaided OHI listeners showed much larger elevations in consonant-identification thresholds than in SeRTs. For Group A consonants, thresholds were elevated by 23.9 dB, while for Group B and Group C consonants, the threshold elevations were 30.2 and 59.1 dB. In contrast, SeRTs were elevated by 6.9 dB. Several factors may help explain the large dissociation between SeRTs and consonant-identification thresholds. First, vowel and intonation cue processing is relatively preserved and provides information about the temporal structure of sentences. Second, OHI listeners can extract partial information about consonants at SNRs below their consonant-identification thresholds. For example, our CaST results suggest that OHI listeners perceive consonant-voicing cues about as accurately as ONH listeners. Such partial consonant information can facilitate the understanding of words, phrases, and sentences in high-context situations, but provides little help in identifying consonants in isolated nonsense syllables. Finally, the average SNRs of individual syllables in HINT and QSIN sentences decline by 6–10 dB over the course of the sentence [41]: i.e., consonants that occur early in sentences are presented at higher SNRs than those that occur later. This may permit OHI listeners to identify some consonants early in the sentence that provide context for words occurring later.

While hearing loss substantially impairs consonant identification, its influence on sentence perception is substantially reduced in high-context sentences [31] relative to more complex material [73]. HINT and QSIN sentences benefit from substantial top-down semantic processing because the sentences are short and have simple syntactic structure and vocabulary. For example, the length of HINT sentences is highly constrained (sentences range from 6 to 8 syllables in length), with 58% of sentences having a simple declarative structure of the form “The cat caught a little mouse”. HINT word selection is also constrained with 22% of the sentences containing only monosyllabic words, and most of the remaining sentences containing only a single two-syllable word. As a result, top-down semantic processing can more effectively compensate for poor consonant identification to a much greater extent than when listening to more complex material, such as sermons, lectures, or film dialogs.

Consonant-identification deficits among aided and unaided OHI listeners were greater for Group B and Group C consonants than for Group A consonants, and Group B and Group C thresholds were far above the SNRs used for HINT and QSIN testing. Nevertheless, Group B and Group C consonants account for more than 60% of consonant utterances in natural speech, suggesting that their identification plays a critical role in the comprehension of more complex listening material where top-down semantic processing is less able to compensate for poor consonant identification.

HAs produced much larger and more consistent improvements on consonant-identification thresholds than on SeRTs (17.7 dB vs. 2.0 dB), in part because HA benefit on SeRTs was limited by the relatively small SeRT elevations in unaided conditions. Indeed, HAs produced significant improvements in consonant-identification thresholds in 83% of OHI listeners, but significant improvements in SeRTs in only 38%. Previous studies have also found greater HA benefit on less semantically constrained materials; for example, greater HA benefit on word lists than on sentences tests [28,79]. Thus, while the mean HA benefit on SeRTs (2.0 dB) was accurately predicted by improvements in Group A consonant thresholds, it was only a small fraction of the mean HA benefit (15.5 dB) measured for Group A consonants.

## Conclusions

We found that 96% of OHI listeners with mild to moderately severe hearing loss showed significant elevations in consonant-identification thresholds. Hearing aids significantly improved the identification thresholds of all consonants, and produced statistically significant improvements in consonant-identification thresholds in more than 80% of OHI listeners. In contrast, SeRT measures showed small and variable elevations in unaided OHI listeners, and inconsistent HA benefit. Importantly, SeRTs in unaided listeners were accurately predicted by the thresholds of easily identified consonants, and HA benefit on SeRTs was similarly predicted by HA benefit on easily identified consonants. Our results suggest that consonant-identification tests provide more detailed and precise assessments of speech-processing deficits and HA benefits in OHI listeners than do SeRTs.

## Supporting Information

S1 Fig Consonant group thresholds. Consonant group thresholds for ONH listeners (blue), and OHI listeners in unaided (green) and aided (red) listening conditions. Error bars show standard error.
(TIF)

S2 Fig Percentage of consonant thresholds in the normal range. Percentage of OHI listeners with consonant-identification thresholds in the ONH range in unaided (green) and aided (red) listening conditions. Asterisks mark consonants where at least 40% of aided OHI were in the ONH range. Minus signs mark consonants where less than 10% were in that range
(TIF)

S3 Fig Psychometric slopes. Slopes of psychometric functions for each onset and coda consonant for OHI listeners, unaided (Panel A) and aided (Panel B), plotted versus the same slopes for ONH listeners for onset (yellow) and coda (purple) consonants.
(TIF)

S4 Fig The MPTA, consonant thresholds, and HA benefit. Scatter plots showing the relationship between the mean MPTA for OHI listeners and their unaided consonant identification thresholds (Panel A) and HA benefit (Panel B).
(TIF)

S5 Fig HINT and QSIN SeRTs. Average SeRTs for ONH, and unaided (green) and aided (red) OHI listeners. Error bars show standard error.
(TIF)

S1 Table Listener age and hearing aid characteristics. Type of hearing aid, listener age, attack and release times and frequency compression settings for the bilateral hearing aids used by each of the 24 listeners.
(DOCX)

S2 Table Consonant presentation SNRs. Mean baseline (B) SNRs used for consonants in onset and coda positions in ONH listeners and average B values used for OHI listeners.
(DOCX)

S3 Table Correlations of pure-tone thresholds and unaided consonant group thresholds, P/I slopes, and SeRTs. Correlations between audiometric thresholds at different frequencies and Groups A, B, and C consonant thresholds in unaided conditions, all consonants combined, psychometric slopes, and SeRTs (discussed in Exp 2). PTA = 0.5, 1 and 2 kHz, MPTA = 1, 2, and 3 kHz; HPTA = 3, 4, 6, and 8 kHz.
(DOCX)

S4 Table Correlations of pure-tone thresholds and unaided thresholds for individual consonants. Correlations of listeners’ pure tone thresholds with their unaided consonant thresholds, averaged over consonant position (except for /ŋ/ and /h/). Type font indicates significance: **<0.001**, **<0.005**, <0.01, *<0.02*.
(DOCX)

S5 Table Hearing aid benefit for individual consonants. Unaided and aided SNR thresholds of OHI listeners for each consonant, along with the results of the ANOVA on the Aided-Unaided difference. Average thresholds are given in dB SNR. Average dB SNR thresholds for ONH listeners (Woods et al., 2012) are given in parentheses in the first column for comparison.
(DOCX)

S6 Table Confusion matrices for onset (S6a) and coda (S6b) consonants in unaided listening conditions. (DOCX)

S7 Table Confusion matrices for onset (S7a) and coda (S7b) consonants in aided listening conditions. (DOCX)

S8 Table Correlations of pure-tone thresholds and aided thresholds for individual consonants. Averaged over consonant position (except for /ŋ/ and /h/). Type font indicates significance: **<0.001**, **<0.005**, <0.01, *<0.02*.
(DOCX)

S9 Table Correlations of pure-tone audiometric thresholds (averaged over ears) with HA benefit on consonant group thresholds and on SeRTs. (DOCX)
