# Supplementary figures and images for: Aided and Unaided Speech Perception by Older Hearing Impaired Listeners
Source: PLoS One. 2015 Mar 2;10(3):e0114922. doi: 10.1371/journal.pone.0114922 (PMC4346396; doi:10.1371/journal.pone.0114922)

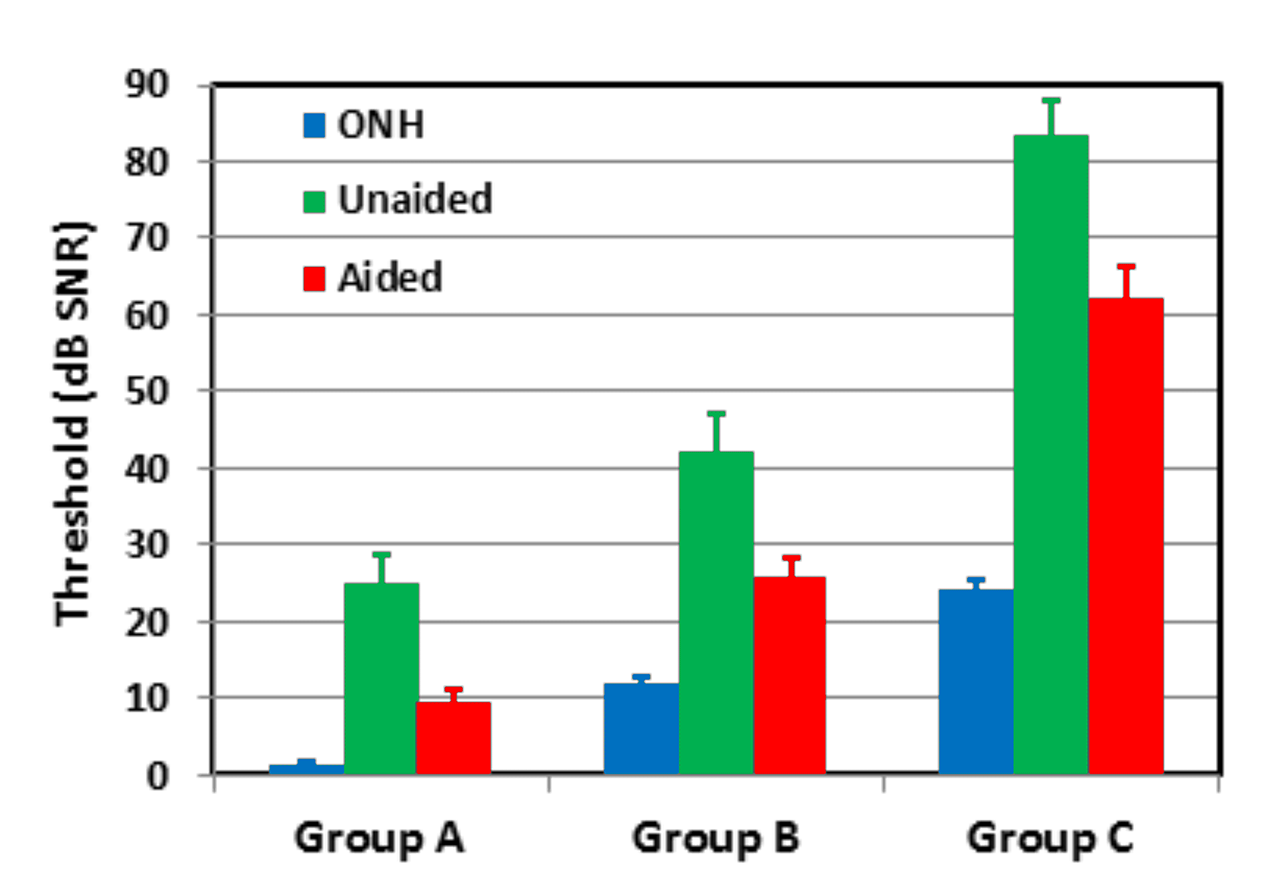

Supplement: S1 Fig — Consonant group thresholds for ONH listeners (blue), and OHI listeners in unaided (green) and aided (red) listening conditions. Error bars show standard error. (TIF) [file pone.0114922.s001.tif]

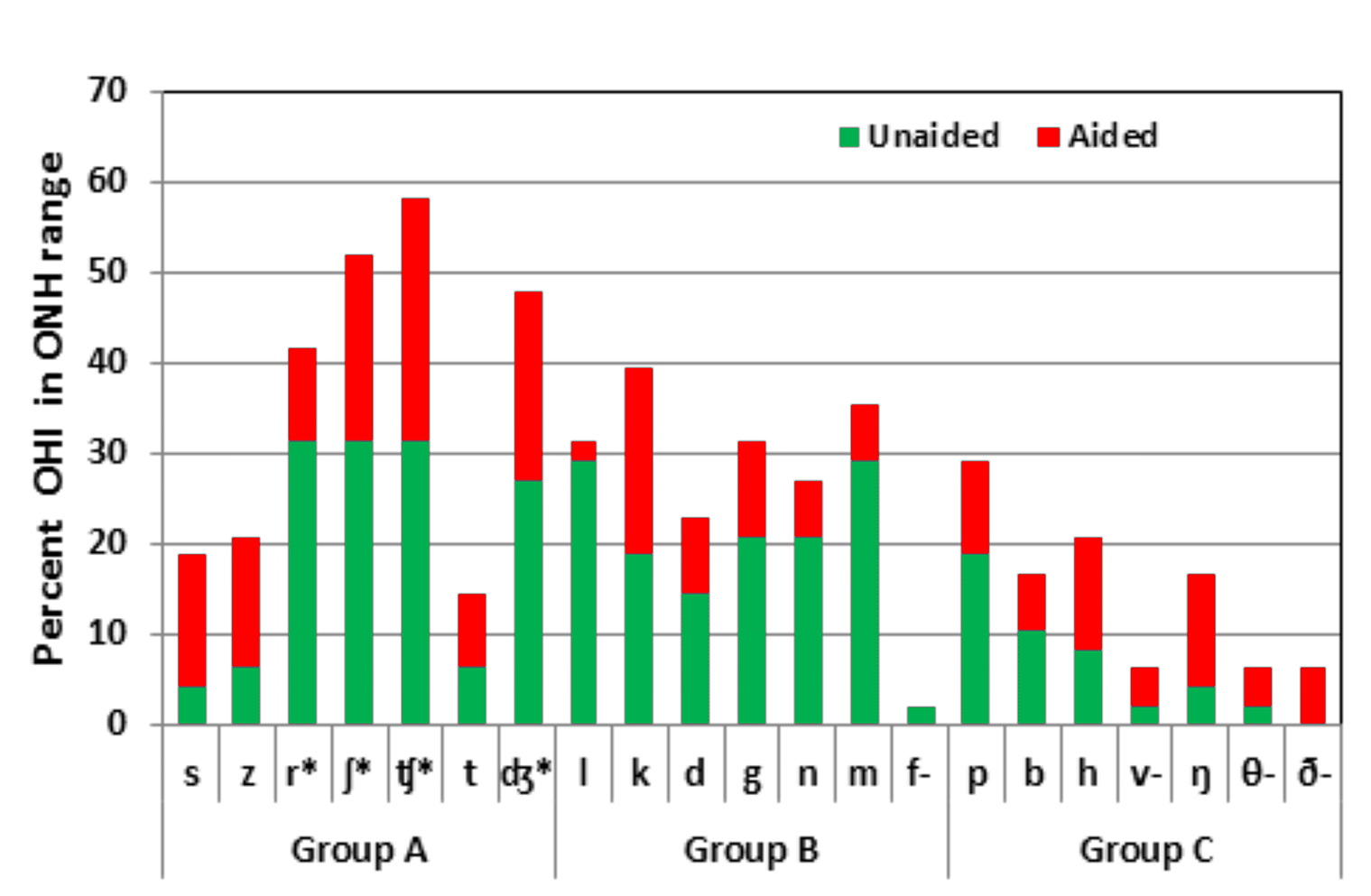

Supplement: S2 Fig — Percentage of OHI listeners with consonant-identification thresholds in the ONH range in unaided (green) and aided (red) listening conditions. Asterisks mark consonants where at least 40% of aided OHI were in the ONH range. Minus signs mark consonants where less than 10% were in that range (TIF) [file pone.0114922.s002.tif]

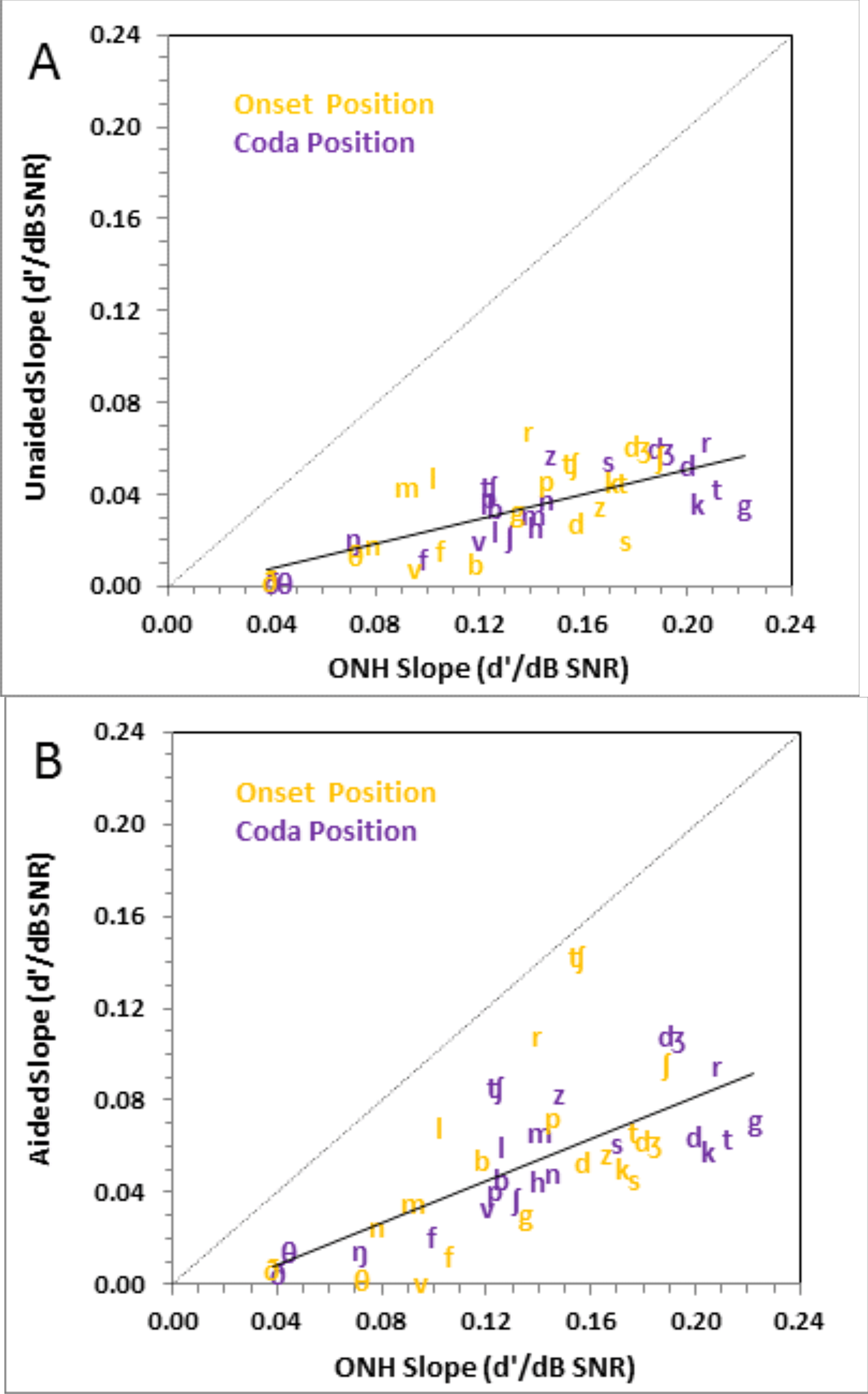

Supplement: S3 Fig — Slopes of psychometric functions for each onset and coda consonant for OHI listeners, unaided (Panel A) and aided (Panel B), plotted versus the same slopes for ONH listeners for onset (yellow) and coda (purple) consonants. (TIF) [file pone.0114922.s003.tif]

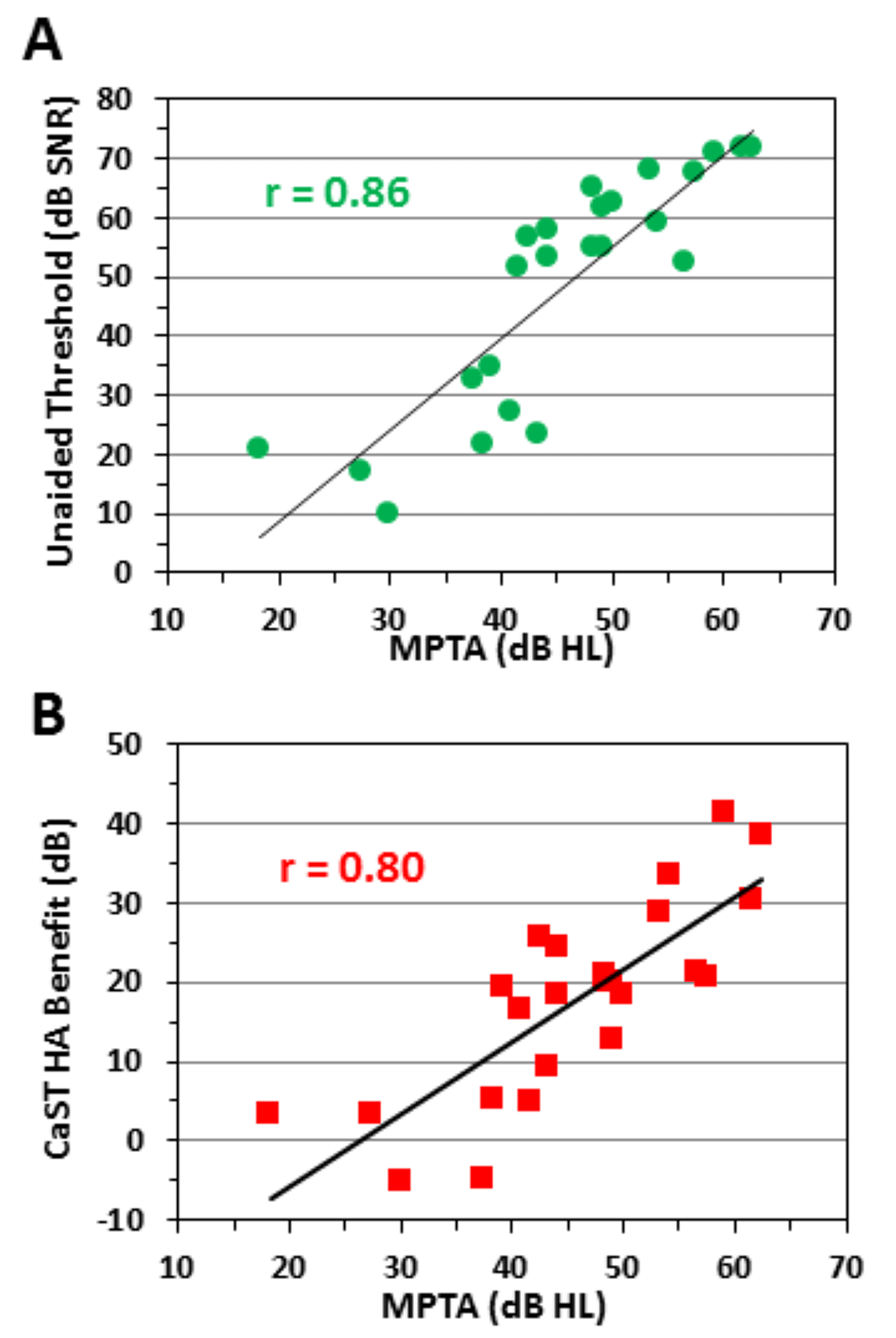

Supplement: S4 Fig — Scatter plots showing the relationship between the mean MPTA for OHI listeners and their unaided consonant identification thresholds (Panel A) and HA benefit (Panel B). (TIF) [file pone.0114922.s004.tif]

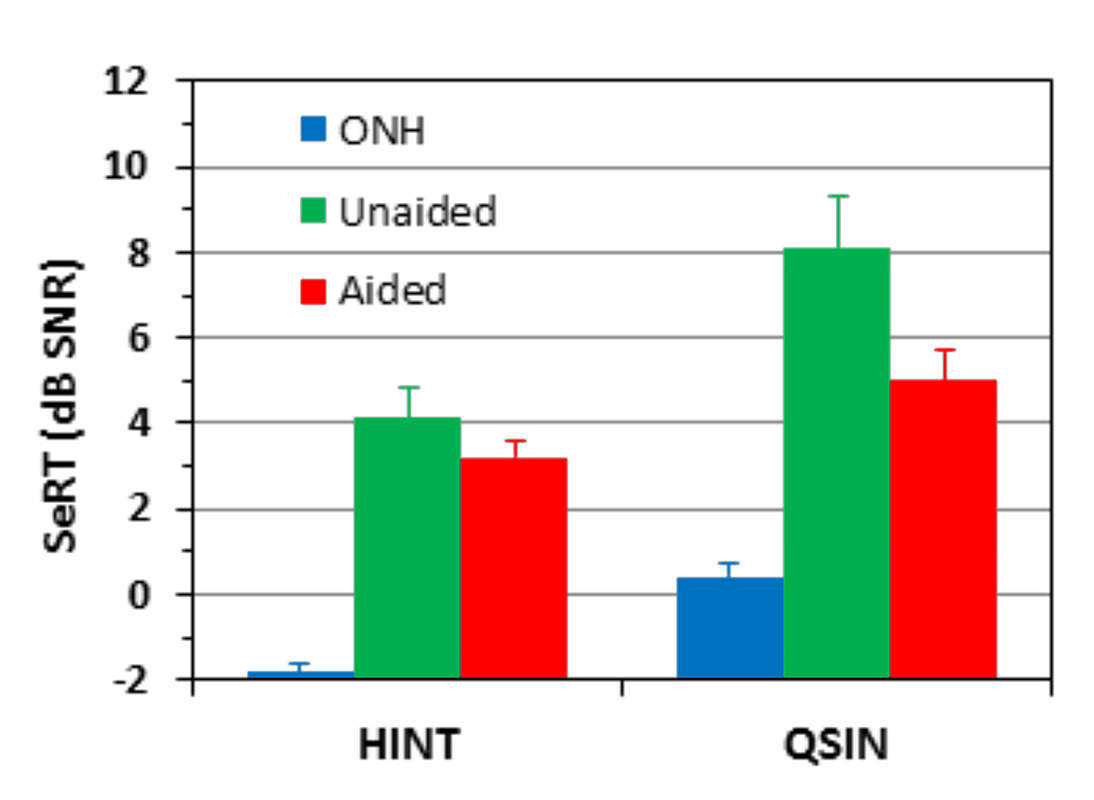

Supplement: S5 Fig — Average SeRTs for ONH, and unaided (green) and aided (red) OHI listeners. Error bars show standard error. (TIF) [file pone.0114922.s005.tif]
