# Supplementary material for: Aided and Unaided Speech Perception by Older Hearing Impaired Listeners
Source: PLoS One. 2015 Mar 2;10(3):e0114922. doi: 10.1371/journal.pone.0114922 (PMC4346396; doi:10.1371/journal.pone.0114922)
Supplement: S1 Table — Type of hearing aid, listener age, attack and release times and frequency compression settings for the bilateral hearing aids used by each of the 24 listeners. (DOCX) [file pone.0114922.s006.docx]

| Listener | HA model | Age | Attack/ release times | Frequency transposition/lowering |
| --- | --- | --- | --- | --- |
| 1 | Phonak Exelia VZ | 69 | 1/50 | no |
| 2 | Phonak Exelia Art Micro BTE | 61 | 1/50 | no |
| 3 | Phonak Ambra | 67 | 10/50 | 3.8 kHz |
| 4 | Phonak Micro Ambra M BTE | 71 | 1/50 | 4.54 kHz |
| 5 | Phonak Exelia Art HS | 78 | 1/50 | no |
| 6 | Phonak Ambra canal | 71 | 10/50 | 4.5 kHz |
| 7 | Phonak Audeo Spice Smart BTE | 64 | 10/50 | no |
| 8 | GN Resound micro BTE RIC | 61 | 12/70 | no |
| 9 | Phonak Ambra CIC | 71 | 10/50 | 4.4 kHz |
| 10 | GN Resound CIC w/mic-in the helix | 61 | 12/70 | no |
| 11 | Phonak Exelia Art M VZ BTE | 66 | 1/50 | 3.7 kHz |
| 12 | Phonak Ambra Micro M BTE | 64 | 1/50 | no |
| 13 | Phonak Audeo Smart Micro BTE | 77 | 1/50 | 3.3 kHz |
| 14 | Phonak Audeo Smart mini BTE RIC | 75 | 1/50 | 3.6 kHz |
| 15 | Phonak Ambra CA | 67 | 10/50 | no |
| 16 | Phonak Exelia Art M | 71 | 1/50 | 3.8 kHz |
| 17 | Phonak Ambra M | 63 | 1/50 | no |
| 18 | Phonak Audeo Mini BTE RIC | 66 | 1/50 | 3.9 kHz |
| 19 | Phonak Ambra CA | 79 | 1/10 | no |
| 20 | Phonak Micro Exelia Art BTE | 78 | 1/50 | 3.8 kHz |
| 21 | Phonak Ambra M BTE | 72 | 1/10 | no |
| 22 | Phonak Exelia Art M VZ BTE | 78 | 1/50 | 3.8 kHz |
| 23 | Phonak Exelia Art | 69 | 1/50 | 4.2 kHz |
| 24 | Phonak Ambra Micro M | 81 | 1/50 | no |
