# Supplementary material for: Aided and Unaided Speech Perception by Older Hearing Impaired Listeners
Source: PLoS One. 2015 Mar 2;10(3):e0114922. doi: 10.1371/journal.pone.0114922 (PMC4346396; doi:10.1371/journal.pone.0114922)
Supplement: S2 Table — Mean baseline (B) SNRs used for consonants in onset and coda positions in ONH listeners and average B values used for OHI listeners. (DOCX) [file pone.0114922.s007.docx]

|  | Onset | | Coda | |
| --- | --- | --- | --- | --- |
| Consonant | ONH | OHI | ONH | OHI |
| s | -4.4 | 7.7 | -4.9 | 17.1 |
| z | 0.4 | 10.9 | 1.2 | 13.5 |
| r | 5.5 | 10.6 | 2.7 | 4.4 |
| ʃ | 4.1 | 9.3 | 3.3 | 10.2 |
| ʧ | 3.9 | 6.5 | 3.4 | 6.2 |
| t | 1.5 | 12.0 | 6.2 | 17.2 |
| ʤ | 2.6 | 6.4 | 7.5 | 14.1 |
| l | 13.5 | 20.6 | 7.4 | 10.9 |
| k | 9.9 | 14.8 | 10.5 | 17.3 |
| d | 6.8 | 13.7 | 14.6 | 24.9 |
| g | 9.9 | 13.8 | 16.8 | 28.8 |
| n | 8.7 | 13.7 | 20.5 | 28.0 |
| m | 10.8 | 16.2 | 20.5 | 25.8 |
| f | 12.1 | 28.8 | 14.1 | 31.7 |
| p | 15.7 | 19.5 | 15.3 | 24.5 |
| b | 13.3 | 23.8 | 16.0 | 25.6 |
| h | 18.5 | 24.0 | - | - |
| v | 13.6 | 29.7 | 24.4 | 38.5 |
| ŋ | - | - | 22.3 | 30.1 |
| ɵ | 22.7 | 38.3 | 20.6 | 38.0 |
| ð | 22.6 | 38.3 | 35.6 | 40.0 |
