# Supplementary material for: Aided and Unaided Speech Perception by Older Hearing Impaired Listeners
Source: PLoS One. 2015 Mar 2;10(3):e0114922. doi: 10.1371/journal.pone.0114922 (PMC4346396; doi:10.1371/journal.pone.0114922)
Supplement: S3 Table — Correlations between audiometric thresholds at different frequencies and Groups A, B, and C consonant thresholds in unaided conditions, all consonants combined, psychometric slopes, and SeRTs (discussed in Exp 2). PTA = 0.5, 1 and 2 kHz, MPTA = 1, 2, and 3 kHz; HPTA = 3, 4, 6, and 8 kHz. (DOCX) [file pone.0114922.s008.docx]

|  | Group A | Group B | Group C | CaST Mean | CaST P/S Slope | SeRT |
| --- | --- | --- | --- | --- | --- | --- |
| 250 | 0.05 | 0.20 | 0.07 | 0.12 | -0.35 | 0.12 |
| 500 | 0.24 | 0.49 | 0.33 | 0.38 | -0.56 | 0.26 |
| 1000 | 0.50 | 0.71 | 0.57 | 0.63 | -0.71 | 0.41 |
| 2000 | 0.80 | 0.85 | 0.81 | 0.86 | -0.71 | 0.81 |
| 3000 | 0.63 | 0.54 | 0.50 | 0.58 | -0.33 | 0.65 |
| 4000 | 0.54 | 0.30 | 0.20 | 0.36 | -0.14 | 0.48 |
| 6000 | 0.40 | 0.19 | 0.22 | 0.28 | -0.03 | 0.35 |
| 8000 | 0.31 | 0.23 | 0.28 | 0.28 | -0.75 | 0.20 |
| PTA | 0.62 | 0.82 | 0.69 | 0.75 | -0.78 | 0.60 |
| MPTA | 0.79 | 0.88 | 0.78 | 0.86 | -0.74 | 0.77 |
| HPTA | 0.58 | 0.40 | 0.39 | 0.48 | -0.21 | 0.53 |
