# Supplementary material for: Aided and Unaided Speech Perception by Older Hearing Impaired Listeners
Source: PLoS One. 2015 Mar 2;10(3):e0114922. doi: 10.1371/journal.pone.0114922 (PMC4346396; doi:10.1371/journal.pone.0114922)
Supplement: S4 Table — Correlations of listeners’ pure tone thresholds with their unaided consonant thresholds, averaged over consonant position (except for /ŋ/ and /h/). Type font indicates significance: <0.001, <0.005, <0.01, <0.02. (DOCX) [file pone.0114922.s009.docx]

| Unaided Consonant | Frequency (Hz) | | | | | | |
| --- | --- | --- | --- | --- | --- | --- | --- |
|  | 500 | 1000 | 2000 | 3000 | 4000 | 6000 | 8000 |
| b |  | **0.63** | **0.80** | *0.47* |  |  |  |
| d | *0.47* | **0.69** | **0.78** | 0.52 |  |  |  |
| g | 0.52 | **0.69** | **0.83** | 0.48 |  |  |  |
| r |  | **0.68** | **0.85** | **0.55** |  |  |  |
| l | **0.55** | **0.73** | **0.85** | 0.51 |  |  |  |
| ŋ |  | *0.47* | **0.78** |  |  |  |  |
| n | *0.43* | **0.62** | **0.77** | **0.57** |  |  |  |
| m | **0.58** | **0.70** | **0.79** | 0.52 |  |  |  |
| v |  | **0.59** | **0.83** | **0.53** |  |  |  |
| ð |  |  | 0.50 |  |  |  |  |
| z |  | *0.44* | **0.68** | 0.50 | 0.48 | *0.47* |  |
| ʤ |  | *0.47* | **0.81** | **0.65** | *0.47* |  |  |
| ʧ |  |  | **0.77** | **0.70** | **0.58** |  |  |
| ʃ |  |  | **0.71** | **0.68** | **0.57** |  |  |
| s |  |  | **0.58** | *0.46* | **0.63** | **0.60** |  |
| θ |  | 0.48 | *0.47* |  |  |  | **0.55** |
| f | **0.53** | **0.73** | **0.86** | **0.53** |  |  |  |
| p |  | **0.60** | **0.82** | **0.53** |  |  |  |
| t |  | **0.54** | **0.75** | **0.55** | *0.43* |  |  |
| k |  | **0.60** | **0.87** | **0.57** |  |  |  |
| h |  | 0.49 | **0.77** | 0.51 |  |  |  |
