# Supplementary material for: Aided and Unaided Speech Perception by Older Hearing Impaired Listeners
Source: PLoS One. 2015 Mar 2;10(3):e0114922. doi: 10.1371/journal.pone.0114922 (PMC4346396; doi:10.1371/journal.pone.0114922)
Supplement: S5 Table — Unaided and aided SNR thresholds of OHI listeners for each consonant, along with the results of the ANOVA on the Aided-Unaided difference. Average thresholds are given in dB SNR. Average dB SNR thresholds for ONH listeners (Woods et al., 2012) are given in parentheses in the first column for comparison. (DOCX) [file pone.0114922.s010.docx]

| Consonant (ONH) | Unaided threshold | Aided threshold | F[1,23] | *p* |
| --- | --- | --- | --- | --- |
| Group A |  |  |  |  |
| s (-4.3) | 35.8 | 15.8 | 42.17 | 0.0000 |
| z (0.8) | 31.9 | 14.1 | 49.71 | 0.0000 |
| r (1.1) | 15.4 | 6.3 | 12.77 | 0.0016 |
| ʃ (1.4) | 18.6 | 4.6 | 12.35 | 0.0019 |
| ʧ (2.2) | 15.6 | 3.3 | 15.33 | 0.0007 |
| t (3.2) | 37.2 | 17.3 | 46.39 | 0.0000 |
| ʤ (3.2) | 21.7 | 6.3 | 16.74 | 0.0004 |
| Group B |  |  |  |  |
| l (8.9) | 27.9 | 19.1 | 10.04 | 0.0043 |
| k (9.2) | 40.2 | 15.9 | 28.24 | 0.0000 |
| d (9.9) | 47.8 | 25.6 | 27.64 | 0.0000 |
| g (13.1) | 46.8 | 27.0 | 31.34 | 0.0000 |
| n (13.5) | 38.4 | 25.0 | 16.13 | 0.0005 |
| m (13.7) | 33.1 | 22.9 | 12.37 | 0.0018 |
| f (15.9) | 63.4 | 44.8 | 53.14 | 0.0000 |
| Group C |  |  |  |  |
| p (14.6) | 68.0 | 35.0 | 27.50 | 0.0000 |
| b (16.5) | 75.2 | 50.8 | 28.07 | 0.0000 |
| h (18.5) | 79.1 | 49.5 | 24.73 | 0.0000 |
| v (21.2) | 84.1 | 64.7 | 52.87 | 0.0000 |
| ŋ (25.6) | 78.8 | 55.8 | 32.93 | 0.0000 |
| θ (26.2) | 95.2 | 80.6 | 14.94 | 0.0008 |
| ð (44.6) | 98.6 | 88.6 | 13.23 | 0.0014 |
