# Supplementary material for: Aided and Unaided Speech Perception by Older Hearing Impaired Listeners
Source: PLoS One. 2015 Mar 2;10(3):e0114922. doi: 10.1371/journal.pone.0114922 (PMC4346396; doi:10.1371/journal.pone.0114922)
Supplement: S6 Table — (DOCX) [file pone.0114922.s011.docx]

|  | b | d | g | r | l | n | m | v | ð | z | ʤ | ʧ | ʃ | s | θ | f | p | t | k | h |
| --- | --- | --- | --- | --- | --- | --- | --- | --- | --- | --- | --- | --- | --- | --- | --- | --- | --- | --- | --- | --- |
| b | 268 | 28 | 9 | 7 | 31 | 2 | 16 | 116 | 2 | 10 | 3 | 2 | 4 | 47 | 25 | 136 | 13 | 13 | 9 | 123 |
| d | 45 | 329 | 30 | 5 | 16 | 6 | 8 | 37 | 10 | 44 | 12 | 5 | 17 | 91 | 29 | 37 | 14 | 33 | 11 | 85 |
| g | 36 | 75 | 318 | 15 | 29 | 8 | 11 | 52 | 3 | 26 | 20 | 3 | 12 | 54 | 8 | 33 | 15 | 8 | 31 | 107 |
| r | 17 | 12 | 10 | 425 | 157 | 8 | 22 | 71 | 5 | 35 | 7 | 2 | 6 | 19 | 5 | 16 | 2 | 6 | 4 | 35 |
| l | 21 | 10 | 1 | 35 | 629 | 12 | 42 | 47 | 5 | 11 | 2 | 1 | 3 | 9 | 1 | 8 | 0 | 3 | 1 | 23 |
| n | 9 | 4 | 4 | 21 | 191 | 352 | 154 | 33 | 4 | 13 | 1 | 1 | 2 | 8 | 1 | 6 | 1 | 5 | 3 | 51 |
| m | 18 | 2 | 1 | 21 | 137 | 81 | 505 | 28 | 1 | 5 | 3 | 0 | 1 | 5 | 2 | 5 | 3 | 1 | 0 | 45 |
| v | 62 | 19 | 11 | 42 | 79 | 3 | 23 | 471 | 25 | 64 | 4 | 0 | 0 | 10 | 9 | 21 | 2 | 5 | 0 | 14 |
| ð | 68 | 76 | 11 | 24 | 134 | 10 | 11 | 227 | 68 | 128 | 2 | 0 | 1 | 12 | 21 | 22 | 3 | 7 | 3 | 36 |
| z | 24 | 52 | 21 | 60 | 80 | 8 | 8 | 81 | 14 | 333 | 35 | 3 | 6 | 42 | 10 | 19 | 2 | 17 | 5 | 44 |
| ʤ | 11 | 56 | 14 | 7 | 31 | 2 | 8 | 31 | 4 | 34 | 396 | 94 | 18 | 25 | 8 | 8 | 7 | 34 | 17 | 59 |
| ʧ | 4 | 6 | 3 | 1 | 8 | 1 | 2 | 6 | 2 | 6 | 59 | 479 | 50 | 59 | 7 | 19 | 6 | 82 | 21 | 43 |
| ʃ | 2 | 11 | 8 | 0 | 2 | 0 | 0 | 2 | 0 | 17 | 51 | 221 | 330 | 81 | 12 | 26 | 6 | 30 | 17 | 48 |
| s | 17 | 20 | 8 | 4 | 15 | 4 | 2 | 16 | 3 | 95 | 9 | 27 | 29 | 346 | 26 | 69 | 20 | 56 | 27 | 71 |
| θ | 33 | 17 | 5 | 5 | 15 | 0 | 3 | 23 | 9 | 26 | 0 | 4 | 9 | 191 | 138 | 244 | 27 | 22 | 14 | 79 |
| f | 44 | 8 | 4 | 2 | 7 | 1 | 4 | 28 | 1 | 11 | 0 | 7 | 12 | 108 | 45 | 395 | 29 | 21 | 11 | 126 |
| p | 14 | 3 | 5 | 3 | 10 | 7 | 5 | 10 | 1 | 3 | 3 | 14 | 3 | 32 | 11 | 45 | 330 | 75 | 88 | 202 |
| t | 10 | 10 | 7 | 3 | 10 | 1 | 1 | 18 | 1 | 12 | 6 | 32 | 19 | 54 | 16 | 46 | 51 | 323 | 82 | 162 |
| k | 12 | 8 | 9 | 3 | 10 | 0 | 6 | 6 | 1 | 5 | 4 | 15 | 6 | 37 | 8 | 33 | 55 | 83 | 410 | 153 |
| h | 12 | 6 | 2 | 1 | 10 | 1 | 10 | 9 | 0 | 6 | 1 | 6 | 14 | 31 | 11 | 53 | 89 | 45 | 69 | 488 |

Table S6a. Confusion matrix for onset consonants in unaided listening conditions.

|  | b | d | g | r | l | ŋ | n | m | v | ð | z | ʤ | ʧ | ʃ | s | θ | f | p | t | k |
| --- | --- | --- | --- | --- | --- | --- | --- | --- | --- | --- | --- | --- | --- | --- | --- | --- | --- | --- | --- | --- |
| b | 454 | 189 | 27 | 2 | 4 | 1 | 7 | 10 | 87 | 14 | 15 | 6 | 1 | 1 | 8 | 9 | 7 | 15 | 6 | 1 |
| d | 91 | 568 | 21 | 6 | 6 | 2 | 9 | 8 | 55 | 36 | 11 | 9 | 1 | 1 | 5 | 11 | 5 | 6 | 12 | 1 |
| g | 106 | 234 | 299 | 7 | 11 | 2 | 6 | 11 | 90 | 24 | 19 | 5 | 2 | 1 | 0 | 9 | 2 | 12 | 19 | 5 |
| r | 15 | 46 | 25 | 397 | 103 | 10 | 30 | 25 | 78 | 11 | 30 | 14 | 8 | 12 | 11 | 6 | 8 | 7 | 21 | 7 |
| l | 25 | 26 | 20 | 72 | 514 | 12 | 17 | 26 | 101 | 4 | 11 | 3 | 0 | 2 | 6 | 3 | 6 | 3 | 10 | 3 |
| ŋ | 9 | 28 | 2 | 17 | 35 | 215 | 238 | 181 | 64 | 18 | 34 | 6 | 1 | 1 | 3 | 6 | 1 | 2 | 2 | 1 |
| n | 10 | 38 | 2 | 14 | 25 | 32 | 489 | 147 | 59 | 11 | 25 | 4 | 2 | 1 | 1 | 1 | 0 | 0 | 3 | 0 |
| m | 25 | 21 | 4 | 6 | 18 | 26 | 156 | 519 | 43 | 7 | 20 | 5 | 0 | 0 | 1 | 3 | 2 | 3 | 5 | 0 |
| v | 71 | 60 | 31 | 20 | 58 | 10 | 21 | 39 | 431 | 50 | 40 | 6 | 0 | 1 | 4 | 4 | 7 | 3 | 8 | 0 |
| ð | 65 | 169 | 38 | 16 | 47 | 8 | 25 | 24 | 255 | 118 | 59 | 12 | 0 | 0 | 4 | 12 | 6 | 2 | 3 | 1 |
| z | 32 | 86 | 29 | 19 | 41 | 10 | 38 | 25 | 167 | 35 | 289 | 32 | 2 | 10 | 17 | 9 | 9 | 4 | 9 | 1 |
| ʤ | 54 | 160 | 29 | 11 | 12 | 2 | 15 | 9 | 58 | 23 | 30 | 347 | 41 | 9 | 12 | 9 | 15 | 10 | 15 | 3 |
| ʧ | 4 | 11 | 1 | 6 | 1 | 3 | 10 | 9 | 6 | 2 | 4 | 36 | 486 | 28 | 32 | 17 | 31 | 58 | 96 | 23 |
| ʃ | 13 | 18 | 6 | 9 | 3 | 3 | 13 | 7 | 16 | 6 | 11 | 25 | 79 | 417 | 56 | 29 | 53 | 41 | 39 | 20 |
| s | 16 | 28 | 9 | 3 | 11 | 5 | 11 | 9 | 29 | 7 | 19 | 12 | 33 | 55 | 275 | 65 | 87 | 62 | 91 | 37 |
| θ | 19 | 30 | 9 | 7 | 11 | 5 | 5 | 11 | 24 | 5 | 8 | 2 | 19 | 18 | 94 | 212 | 163 | 84 | 104 | 34 |
| f | 13 | 10 | 8 | 5 | 8 | 3 | 7 | 7 | 33 | 2 | 6 | 0 | 8 | 19 | 91 | 109 | 339 | 82 | 73 | 41 |
| p | 38 | 4 | 1 | 6 | 3 | 0 | 0 | 2 | 3 | 1 | 2 | 1 | 15 | 5 | 12 | 30 | 48 | 443 | 154 | 96 |
| t | 10 | 21 | 1 | 4 | 2 | 0 | 5 | 5 | 8 | 1 | 3 | 3 | 40 | 10 | 32 | 48 | 30 | 148 | 407 | 86 |
| k | 9 | 10 | 14 | 5 | 2 | 3 | 6 | 5 | 7 | 1 | 2 | 4 | 24 | 7 | 30 | 36 | 37 | 158 | 138 | 366 |

Table S6b. Confusion matrix for coda consonants in unaided listening conditions.
