# Supplementary material for: Aided and Unaided Speech Perception by Older Hearing Impaired Listeners
Source: PLoS One. 2015 Mar 2;10(3):e0114922. doi: 10.1371/journal.pone.0114922 (PMC4346396; doi:10.1371/journal.pone.0114922)
Supplement: S7 Table — (DOCX) [file pone.0114922.s012.docx]

|  | b | d | g | r | l | n | m | v | ð | z | ʤ | ʧ | ʃ | s | θ | f | p | t | k | h |
| --- | --- | --- | --- | --- | --- | --- | --- | --- | --- | --- | --- | --- | --- | --- | --- | --- | --- | --- | --- | --- |
| b | 332 | 25 | 9 | 11 | 14 | 3 | 12 | 121 | 4 | 1 | 3 | 1 | 1 | 15 | 28 | 149 | 22 | 5 | 7 | 101 |
| d | 47 | 535 | 39 | 6 | 10 | 2 | 3 | 23 | 10 | 16 | 9 | 3 | 6 | 36 | 24 | 14 | 7 | 20 | 11 | 43 |
| g | 33 | 99 | 456 | 13 | 10 | 4 | 12 | 28 | 10 | 12 | 11 | 4 | 2 | 13 | 9 | 22 | 8 | 15 | 35 | 68 |
| r | 16 | 8 | 9 | 596 | 101 | 1 | 13 | 42 | 7 | 15 | 4 | 0 | 3 | 3 | 3 | 12 | 4 | 1 | 2 | 24 |
| l | 21 | 8 | 5 | 37 | 639 | 21 | 35 | 61 | 5 | 2 | 0 | 0 | 1 | 4 | 2 | 4 | 2 | 4 | 1 | 12 |
| n | 7 | 18 | 1 | 18 | 167 | 478 | 99 | 12 | 6 | 8 | 1 | 2 | 3 | 3 | 4 | 3 | 4 | 0 | 3 | 27 |
| m | 15 | 0 | 1 | 13 | 114 | 87 | 556 | 45 | 2 | 2 | 0 | 0 | 1 | 5 | 0 | 4 | 2 | 0 | 1 | 16 |
| v | 65 | 18 | 12 | 62 | 55 | 3 | 16 | 517 | 35 | 35 | 3 | 0 | 0 | 1 | 9 | 16 | 0 | 1 | 1 | 15 |
| ð | 77 | 78 | 8 | 21 | 149 | 7 | 10 | 255 | 105 | 86 | 2 | 0 | 1 | 6 | 20 | 20 | 2 | 2 | 2 | 13 |
| z | 18 | 45 | 15 | 35 | 34 | 8 | 5 | 53 | 14 | 484 | 44 | 1 | 1 | 44 | 5 | 6 | 3 | 12 | 8 | 29 |
| ʤ | 5 | 46 | 12 | 5 | 4 | 0 | 1 | 4 | 3 | 9 | 585 | 104 | 9 | 11 | 2 | 1 | 3 | 40 | 3 | 17 |
| ʧ | 0 | 4 | 2 | 5 | 1 | 2 | 1 | 1 | 0 | 0 | 59 | 666 | 28 | 8 | 2 | 3 | 3 | 68 | 8 | 3 |
| ʃ | 0 | 4 | 0 | 1 | 4 | 0 | 0 | 0 | 0 | 4 | 29 | 279 | 468 | 39 | 0 | 0 | 1 | 18 | 5 | 12 |
| s | 13 | 13 | 8 | 6 | 3 | 3 | 1 | 15 | 5 | 99 | 8 | 24 | 17 | 485 | 19 | 58 | 8 | 37 | 13 | 29 |
| θ | 39 | 18 | 7 | 4 | 10 | 1 | 1 | 16 | 11 | 16 | 3 | 6 | 1 | 175 | 190 | 267 | 19 | 29 | 11 | 40 |
| f | 30 | 7 | 3 | 3 | 10 | 1 | 4 | 25 | 3 | 1 | 1 | 9 | 9 | 81 | 70 | 469 | 30 | 27 | 13 | 68 |
| p | 9 | 3 | 6 | 4 | 5 | 0 | 0 | 6 | 0 | 0 | 2 | 10 | 2 | 6 | 9 | 36 | 469 | 57 | 70 | 170 |
| t | 5 | 9 | 3 | 7 | 3 | 0 | 3 | 4 | 3 | 5 | 7 | 51 | 1 | 17 | 14 | 14 | 52 | 531 | 55 | 80 |
| k | 3 | 4 | 6 | 4 | 3 | 1 | 1 | 4 | 0 | 0 | 7 | 11 | 0 | 23 | 5 | 16 | 42 | 57 | 599 | 78 |
| h | 9 | 0 | 9 | 2 | 5 | 1 | 1 | 7 | 0 | 0 | 2 | 10 | 2 | 7 | 9 | 52 | 74 | 31 | 70 | 573 |

Table 7a. Confusion matrix for onset consonants in aided listening conditions.

|  | b | d | g | r | l | ŋ | n | m | v | ð | z | ʤ | ʧ | ʃ | s | θ | f | p | t | k |
| --- | --- | --- | --- | --- | --- | --- | --- | --- | --- | --- | --- | --- | --- | --- | --- | --- | --- | --- | --- | --- |
| b | 554 | 105 | 27 | 2 | 6 | 0 | 5 | 16 | 93 | 9 | 6 | 0 | 0 | 0 | 1 | 8 | 6 | 24 | 2 | 0 |
| d | 70 | 636 | 28 | 2 | 6 | 1 | 6 | 1 | 40 | 19 | 5 | 8 | 0 | 0 | 2 | 10 | 6 | 5 | 18 | 1 |
| g | 106 | 187 | 411 | 1 | 2 | 1 | 17 | 7 | 56 | 19 | 6 | 4 | 0 | 0 | 0 | 11 | 6 | 12 | 14 | 4 |
| r | 22 | 35 | 27 | 495 | 60 | 9 | 32 | 13 | 53 | 4 | 17 | 25 | 13 | 10 | 9 | 4 | 8 | 7 | 13 | 8 |
| l | 22 | 21 | 12 | 74 | 502 | 19 | 23 | 34 | 109 | 4 | 11 | 9 | 2 | 1 | 2 | 1 | 9 | 5 | 2 | 2 |
| ŋ | 5 | 16 | 8 | 10 | 23 | 286 | 236 | 197 | 51 | 9 | 12 | 2 | 0 | 0 | 2 | 0 | 1 | 0 | 6 | 0 |
| n | 10 | 22 | 5 | 8 | 10 | 40 | 572 | 155 | 27 | 8 | 5 | 0 | 0 | 0 | 2 | 0 | 0 | 0 | 0 | 0 |
| m | 20 | 6 | 0 | 4 | 14 | 41 | 139 | 586 | 29 | 4 | 12 | 1 | 0 | 2 | 0 | 2 | 3 | 1 | 0 | 0 |
| v | 73 | 30 | 18 | 24 | 35 | 9 | 24 | 32 | 536 | 28 | 38 | 3 | 0 | 0 | 1 | 3 | 7 | 3 | 0 | 0 |
| ð | 54 | 126 | 24 | 13 | 17 | 6 | 37 | 24 | 347 | 142 | 57 | 7 | 0 | 0 | 2 | 5 | 1 | 2 | 0 | 0 |
| z | 21 | 34 | 18 | 16 | 8 | 8 | 21 | 13 | 113 | 31 | 477 | 64 | 8 | 4 | 16 | 4 | 5 | 2 | 1 | 0 |
| ʤ | 12 | 75 | 16 | 14 | 3 | 1 | 3 | 2 | 12 | 2 | 13 | 645 | 32 | 7 | 5 | 4 | 3 | 3 | 8 | 4 |
| ʧ | 4 | 1 | 1 | 2 | 0 | 3 | 13 | 4 | 4 | 0 | 3 | 59 | 643 | 39 | 7 | 4 | 7 | 6 | 39 | 25 |
| ʃ | 2 | 6 | 4 | 1 | 0 | 1 | 3 | 2 | 2 | 1 | 3 | 38 | 64 | 668 | 26 | 11 | 6 | 4 | 13 | 9 |
| s | 11 | 6 | 6 | 4 | 1 | 3 | 4 | 1 | 17 | 0 | 21 | 9 | 32 | 75 | 488 | 56 | 63 | 23 | 32 | 12 |
| θ | 11 | 12 | 0 | 10 | 4 | 0 | 0 | 3 | 31 | 8 | 10 | 1 | 6 | 13 | 95 | 285 | 245 | 28 | 72 | 30 |
| f | 7 | 9 | 2 | 4 | 7 | 0 | 4 | 1 | 24 | 0 | 6 | 0 | 2 | 21 | 85 | 118 | 486 | 42 | 31 | 15 |
| p | 41 | 3 | 4 | 0 | 0 | 0 | 1 | 0 | 2 | 0 | 0 | 0 | 6 | 0 | 4 | 28 | 35 | 499 | 135 | 106 |
| t | 5 | 8 | 4 | 0 | 1 | 1 | 1 | 3 | 1 | 0 | 3 | 1 | 26 | 3 | 25 | 36 | 23 | 59 | 601 | 63 |
| k | 5 | 10 | 11 | 0 | 1 | 1 | 3 | 3 | 3 | 1 | 0 | 0 | 15 | 7 | 9 | 21 | 24 | 72 | 109 | 569 |

Table 7b. Confusion matrix for coda consonants in aided listening conditions.
