# Supplementary material for: Aided and Unaided Speech Perception by Older Hearing Impaired Listeners
Source: PLoS One. 2015 Mar 2;10(3):e0114922. doi: 10.1371/journal.pone.0114922 (PMC4346396; doi:10.1371/journal.pone.0114922)
Supplement: S8 Table — Averaged over consonant position (except for /ŋ/ and /h/). Type font indicates significance: <0.001, <0.005, <0.01, <0.02. (DOCX) [file pone.0114922.s013.docx]

| Consonant | Frequency (Hz) | | | | | | |
| --- | --- | --- | --- | --- | --- | --- | --- |
|  | 500 | 1000 | 2000 | 3000 | 4000 | 6000 | 8000 |
| b |  |  | **0.65** | **0.55** |  |  |  |
| d |  |  | **0.57** | 0.51 | 0.50 | **0.54** |  |
| g |  |  | **0.73** | **0.56** |  |  |  |
| r |  |  | **0.65** | *0.45* | *0.44* |  |  |
| l | *0.45* | **0.54** | **0.68** | **0.55** |  |  |  |
| ŋ | *0.45* | **0.64** | **0.63** | 0.50 |  |  |  |
| n |  | *0.44* | **0.69** | *0.46* |  |  |  |
| m |  |  | **0.63** | *0.42* |  |  |  |
| v |  |  | **0.61** | *0.43* |  |  |  |
| ð |  |  | *0.47* | *0.45* | *0.47* | *0.43* |  |
| z |  |  |  |  | *0.42* | **0.53** |  |
| ʤ |  |  |  | *0.44* |  | 0.50 |  |
| ʧ |  |  |  | *0.45* |  | *0.45* |  |
| ʃ |  |  |  | 0.49 | *0.43* | **0.59** | *0.44* |
| s |  |  |  | *0.43* | **0.61** | **0.68** |  |
| θ |  |  |  |  | *0.43* | *0.45* |  |
| f |  | 0.50 | **0.59** |  | *0.43* | *0.46* | 0.50 |
| p |  |  | *0.45* |  |  | **0.53** |  |
| t |  |  |  |  | *0.45* | **0.55** |  |
| k |  |  | **0.53** | *0.46* |  |  |  |
| h |  |  | *0.46* |  |  |  | *0.42* |
