# Supplementary material for: Aided and Unaided Speech Perception by Older Hearing Impaired Listeners
Source: PLoS One. 2015 Mar 2;10(3):e0114922. doi: 10.1371/journal.pone.0114922 (PMC4346396; doi:10.1371/journal.pone.0114922)
Supplement: S9 Table — (DOCX) [file pone.0114922.s014.docx]

|  | Group A HA benefit | Group B HA benefit | Group C HA benefit | Mean CaST HA benefit | SeRT HA benefit |
| --- | --- | --- | --- | --- | --- |
| 250 | 0.02 | 0.21 | 0.12 | 0.13 | -0.03 |
| 500 | 0.23 | 0.54 | 0.33 | 0.43 | 0.07 |
| 1000 | 0.52 | 0.73 | 0.46 | 0.67 | 0.30 |
| 2000 | 0.79 | 0.75 | 0.40 | 0.77 | 0.67 |
| 3000 | 0.54 | 0.43 | 0.07 | 0.43 | 0.54 |
| 4000 | 0.40 | 0.12 | -0.31 | 0.12 | 0.40 |
| 6000 | 0.20 | 0.00 | -0.35 | -0.03 | 0.16 |
| 8000 | 0.53 | 0.73 | 0.43 | 0.66 | 0.36 |
| PTA | 0.62 | 0.80 | 0.47 | 0.75 | 0.43 |
| MPTA | 0.77 | 0.81 | 0.41 | 0.79 | 0.62 |
| HPTA | 0.42 | 0.20 | -0.20 | 0.19 | 0.38 |
